# Antibacterial Hydrogel Adhesives Based on Bifunctional Telechelic Dendritic-Linear–Dendritic Block Copolymers

**DOI:** 10.1021/jacs.4c03673

**Published:** 2024-06-12

**Authors:** Natalia Sanz del Olmo, Noemi Molina, Yanmiao Fan, Faridah Namata, Daniel J. Hutchinson, Michael Malkoch

**Affiliations:** Department of Fibre and Polymer Technology, KTH Royal Institute of Technology, Teknikringen 56-68, 100 44 Stockholm, Sweden

## Abstract

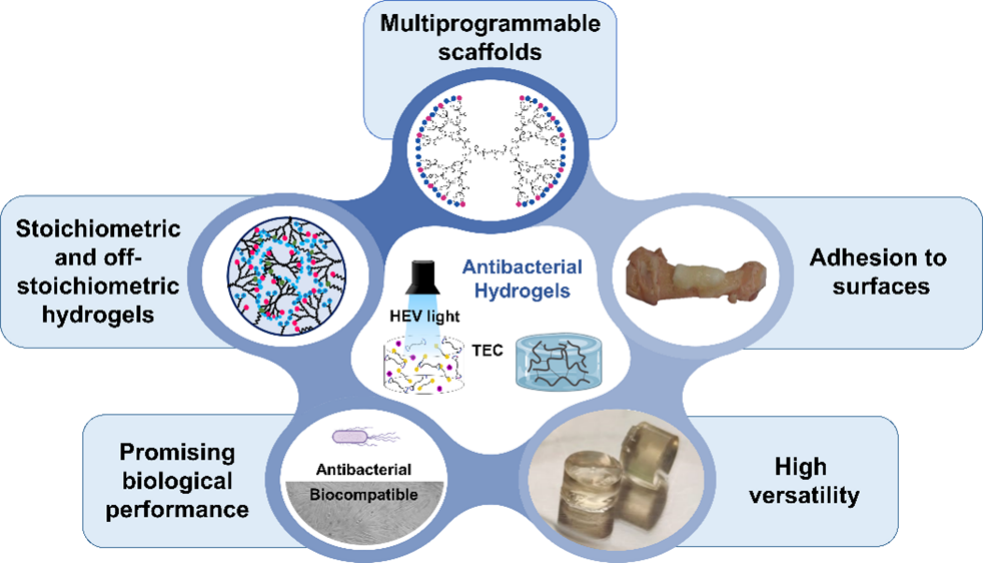

Antibiotic-resistant pathogens have been declared by the WHO as one of the major public health threats facing humanity. For that reason, there is an urgent need for materials with inherent antibacterial activity able to replace the use of antibiotics, and in this context, hydrogels have emerged as a promising strategy. Herein, we introduce the next generation of cationic hydrogels with antibacterial activity and high versatility that can be cured on demand in less than 20 s using thiol–ene click chemistry (TEC) in aqueous conditions. The approach capitalizes on a two-component system: (i) telechelic polyester-based dendritic-linear–dendritic (DLDs) block copolymers of different generations heterofunctionalized with allyl and ammonium groups, as well as (ii) polyethylene glycol (PEG) cross-linkers functionalized with thiol groups. These hydrogels resulted in highly tunable materials where the antibacterial performance can be adjusted by modifying the cross-linking density. Off-stoichiometric hydrogels showed narrow antibacterial activity directed toward Gram-negative bacteria. The presence of pending allyls opens up many possibilities for functionalization with biologically interesting molecules. As a proof-of-concept, hydrophilic cysteamine hydrochloride as well as N-hexyl-4-mercaptobutanamide, as an example of a thiol with a hydrophobic alkyl chain, generated three-component networks. In the case of cysteamine derivatives, a broader antibacterial activity was noted than the two-component networks, inhibiting the growth of Gram-positive bacteria. Additionally, these systems presented high versatility, with storage modulus values ranging from 270 to 7024 Pa and different stability profiles ranging from 1 to 56 days in swelling experiments. Good biocompatibility toward skin cells as well as strong adhesion to multiple surfaces place these hydrogels as interesting alternatives to conventional antibiotics.

## Introduction

The implantation of indwelling bodies has become an essential procedure in most fields of medicine. However, the majority of microorganisms have an extraordinary capacity to adhere to materials and promote biofilm formation as the most relevant feature of their pathogenicity.^1,2^ For that reason, there is an urgent need for antimicrobial biomaterials that can prevent the growth of microorganisms on implants. Hydrogels are promising materials that can be coated on catheters,^3^ contact lenses or dental implants,^4^ used for local injection for drug release,^5^ as coatings to avoid postsurgical site infections (SSIs),^6^ and in wound healing.^7^ However, most of them require the encapsulation of active compounds such as antibiotics or silver ions, and this usually leads to important issues associated with antimicrobial resistance. The misuse and overuse of antibiotics are the main drivers in the development of drug-resistant pathogens. Currently, drug-resistant pathogens are a serious problem declared by the WHO as one of the top ten public health threats facing humanity.^8^ Although antibiotics play a crucial role in modern medicine and have saved innumerable lives, the search for alternatives to conventional antibiotics is of the utmost importance. For that reason, the design of hydrogels expressing intrinsic antibacterial activity is in the spotlight of current research.^9−11^ The most interesting property of hydrogels is their ability to uptake and store many times their weight in water. Considering that most of the human body is water, hydrogels show great potential for use in biomedical applications.^12^ Additionally, their adhesive properties have drawn considerable attention in the field of biomedicine, particularly in the application of wound closure.^13^ As the majority of a hydrogel is composed of water, which does not participate in joining to surfaces, the obtention of materials with strong adhesion is still a great challenge for researchers. Moreover, hydrogels are highly tunable. The versatility of these materials is such that, by a careful selection of the hydrogel′s components, desired properties such as hydrophobicity, porosity, or biomedical activity can be controlled.

Poly(ethylene glycol) (PEG)-based hydrogels have attracted wide interest for their ability to mimic natural extracellular matrices (ECMs) due to their elastic and hydrophilic nature that can be tuned by varying the PEG length or temperature.^14^ Currently, there are examples of PEG-based medical products on the market with outstanding antibacterial performance, where a sophisticated way of introducing multifunctionality to the PEG-based hydrogels has been through the use of dendritic polymers. The inclusion of a hyperbranched poly(ethylenimine) (PEI) cross-linked with *N*-hydroxysuccinimide (NHS) activated PEG resulted in Adherus AutoSpray, a commercially available tissue adhesive with strong antimicrobial activity.^15^ Protonated polyamines have shown great potential with a similar mechanism of action as antimicrobial peptides but at a lower production price.^16^ The described mechanism of action involves multivalent binding between the cations and proteins, which adhere to the bacteria and aggregate on the cell wall, producing a chamber resistance effect to kill the bacteria. The protonated amines can also interact with hydrophilic groups present in the bacteria's membrane, damaging it by changing its permeability.^17^ Previously, our research group reported a nontoxic and hydrolytically degradable antibacterial hydrogel designed to preemptively treat SSIs during the first crucial 24 h period without relying on conventional antibiotics.^6^ The hydrogel was comprised of functional dendritic-linear–dendritic (DLD) hybrids based on linear PEG and dendritic 2,2-bis(hydroxymethyl)propionic acid (bis-MPA) decorated with a precise number of ammonium groups, together with PEG cross-linking containing NHS functional groups. The use of DLDs in the hydrogels, as opposed to hyperbranched polymers, allows for greater structural control and batch-to-batch consistency, including the number of reactive groups. These hydrogels were chemically cross-linked through amidation reactions between the cationic ammonium groups of the DLD and the NHS groups of the cross-linker. While amidation reactions do provide a facile path to hydrogel formation, they have some limitations, such as the release of NHS during the cross-linking reaction. The reaction proceeds spontaneously, which can limit the possibilities for on-tissue formation of the hydrogels. Additionally, the amidation reaction occurs statistically between the amines of the dendritic polymer and the carboxylated cross-linkers, hindering accurate control over the network.

Alternatively, thiol–ene click chemistry (TEC) has been utilized in hydrogel formation. TEC offers higher reproducibility through the obtention of uniform and controllable networks with improved mechanical performances due to the higher stability of the thioether bond.^18^ The reaction proceeds via a rapid, stepwise reaction rate, is highly efficient under mild aqueous conditions, and can be initiated on demand with high-energy visible (HEV) light.^19−21^ Additionally, its high monomer conversion before vitrification reduces the likelihood of unreacted monomers leeching out.^22,23^ As a further step, TEC networks have preclinically been validated in realistic surgical conditions for fracture stabilization.^24^

In this work, we combine the two concepts described previously with the design of heterofunctionalized telechelic DLDs from the second to the fifth generation with an exact number of peripheral antibacterial ammonium groups and allyl functionalities based on 10k and 20k PEGs as linear segments. The high reproducibility of the hydrogels, afforded by the use of both DLDs and the TEC reaction for cross-linking, allows for more precise control of the 3D network and therefore a better opportunity for understanding the impact network properties have on the antibacterial activity of the hydrogels. The ammonium functionalities present on the DLD precursors will provide antibacterial activity; however, the presence of alkene groups will serve as cross-linking points with thiol-functionalized PEGs as cross-linkers via HEV light curing using TEC chemistry. This will help to overcome the drawbacks associated with amidation reactions while keeping the antimicrobial performance. A wide variety of cross-linking densities as well as different weight percentages have been explored and optimized to increase the performance window. Antibacterial activity was screened against Gram-positive and Gram-negative planktonic bacteria strains in order to determine the top candidates for further characterization in terms of mechanical properties and cytotoxicity. Additionally, adhesion to different surfaces has been evaluated and quantified using a new method. Finally, the development of off-stoichiometric TEC gels with an excess of allyl functionalities was used to demonstrate the high versatility of the novel systems by the inclusion of small biologically interesting molecules both hydrophilic and hydrophobic, such as cysteamine hydrochloride (Cys) and N-hexyl-4-mercaptobutanamide (NHMB).

## Materials and Methods

All information related to materials and methods can be found in the Supporting Information.

## Results and Discussion

### Telechelic Block Copolymers with Alternating −[BC_2_]– Heterofunctional Representation

**Figure 1:**
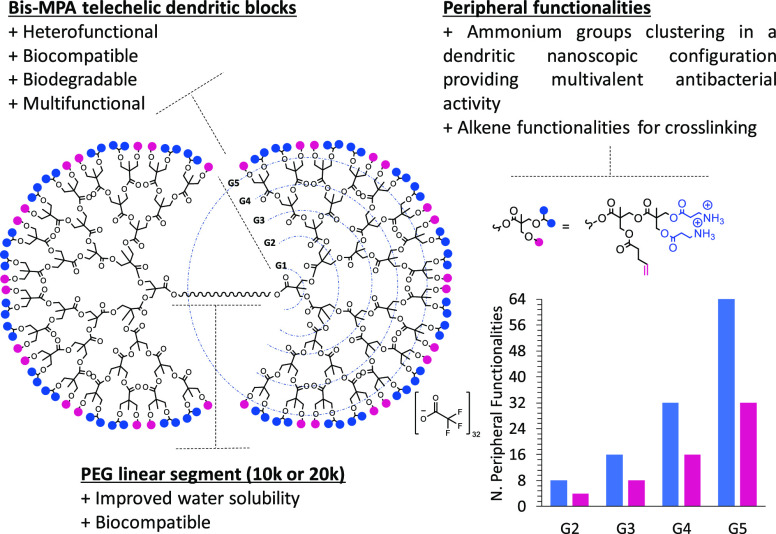
(left) Structure and design features of the fifth-generation PEGXk-G5-(allyl)_32_(NH_3_^+^)_64_ dendritic-linear–dendritic block copolymer (DLD) based on AB_2_C monomer with allyl and ammonium functionalities. (right) Number of allyl and ammonium functionalities present in each of the G2–G5 DLDs.

For the obtention of antibacterial hydrogels, multiprogrammable dendritic-linear–dendritic block copolymers (DLDs) based on bis-MPA were employed. The main characteristic of these highly versatile polyester dendritic scaffolds was their peripheral orthogonal functionalities. Both alkene and ammonium groups were elegantly combined to serve as a source of cross-linking points and antibacterial activity, respectively. Structurally, these systems were based on a linear PEG that provided biocompatibility as well as water solubility to the system. From both ends of the PEG chain, bis-MPA polyester dendrons were grown to different generations with high structural precision. The use of biocompatible bis-MPA building blocks provided the DLDs with biodegradability as well as multivalence. The introduction of allyl and ammonium groups was achieved through the peripheral functionalization of the DLD with the AB_2_C acid monomer. In contrast to linear polymers, the functional groups were displayed on the peripheral of the DLDs in a – ABBA– pattern instead of −ABAB–. The number of peripheral functionalities doubled with each generation, with the fifth generation exhibiting 64 ammonium groups and 32 allyl functionalities (Figure 1). The DLD precursors were prepared via a layer-by-layer divergent growth approach using an iterative combination of anhydride and deprotection chemistries to obtain peripheral hydroxylated dendritic skeletons.^6^ Then, the peripheral groups were esterified with the AB_2_C acid monomer, which was synthesized as previously reported.^25^ For the introduction of the multifunctional monomer, anhydride chemistry was used in the presence of catalytic amounts of 2-(dimethylamine) pyridine (DMAP) and an excess of pyridine. Cationic charges were introduced to the DLD through first deprotecting the peripheral hydroxyl groups with DOWEX, followed by esterification with Boc β-alanine anhydride, and finally deprotection of the β-alanine with trifluoroacetic acid (TFA). This procedure resulted in pure amino-functional DLDs as trifluoroacetate salts (PEG*X*k-G*n*-(allyl)_*m*_(NH_3_^+^)_2*m*_, where *X* = 10 or 20; *n* = 2, 3, 4, 5; and *m* = 4, 8, 16, 32), with yields ranging from 60 to 95%. PEGs of different lengths (10k and 20k) were used, except for the fifth-generation derivative, which was only synthesized with PEG10k due to the higher complexity of its synthesis. All purification steps consisted of simple and straightforward precipitations in cold ether. Even though these scaffolds were prepared with the goal of obtaining antibacterial precursors for the preparation of hydrogels, it is worth highlighting that the dual functionalities can be tuned for other multipurpose studies.

The monitoring of the reactions was conducted using a combination of ^1^H NMR (Figure S1) and ^13^C NMR spectroscopy. The functionalization of the dendritic periphery with the acetonide-protected AB_2_C monomer was confirmed with ^13^C NMR spectroscopy through the shift of the signal from 47.8 to 46.7 ppm, which was attributed to the quaternary carbon of the external bis-MPA moiety before and after esterification. Additionally, by ^1^H NMR spectroscopy, it was possible to observe the presence of the signals attributed to the allyl groups in the range of 5–6 ppm.

The subsequent step of the deprotection of the hydroxyl groups on the AB_2_C functionality was mainly corroborated by ^1^H NMR spectroscopy by the disappearance of the singlets at 1.34 and 1.40 ppm attributed to the methyls of the acetonide-protecting group. Moreover, the complete functionalization of the periphery with Boc β-alanine was confirmed by ^13^C NMR spectroscopy by the disappearance of the signal at 49.8 ppm and the appearance of new signals from the methylene groups in the Boc β-alanine moiety at 3.37 and 2.53 ppm in the ^1^H NMR spectrum as well as 36.5 and 34.2 ppm in the ^13^C NMR spectrum. Finally, the deprotection of the amino functionalities could be clearly confirmed by the disappearance of the Boc signals at 28.5, 79.4, and 155.9 ppm in the ^13^C NMR spectrum and the singlet at 1.44 ppm in the ^1^H NMR spectrum. Size exclusion chromatography (SEC) using DMF as a solvent was utilized to further characterize these dendritic scaffolds. Unfortunately, due to aggregation processes, not all dendritic polymers were suitable for analysis with this technique (Supporting Information). ^1^H NMR spectroscopy showed that the synthesized cationic DLDs were stable in water up to 3 h, confirming their suitability as hydrogel components and suggesting that they would not degrade during hydrogel preparation (Figure S6).

### Two-Component Cationic Hydrogels Based on DLDs-AB_2_C

**Figure 2:**
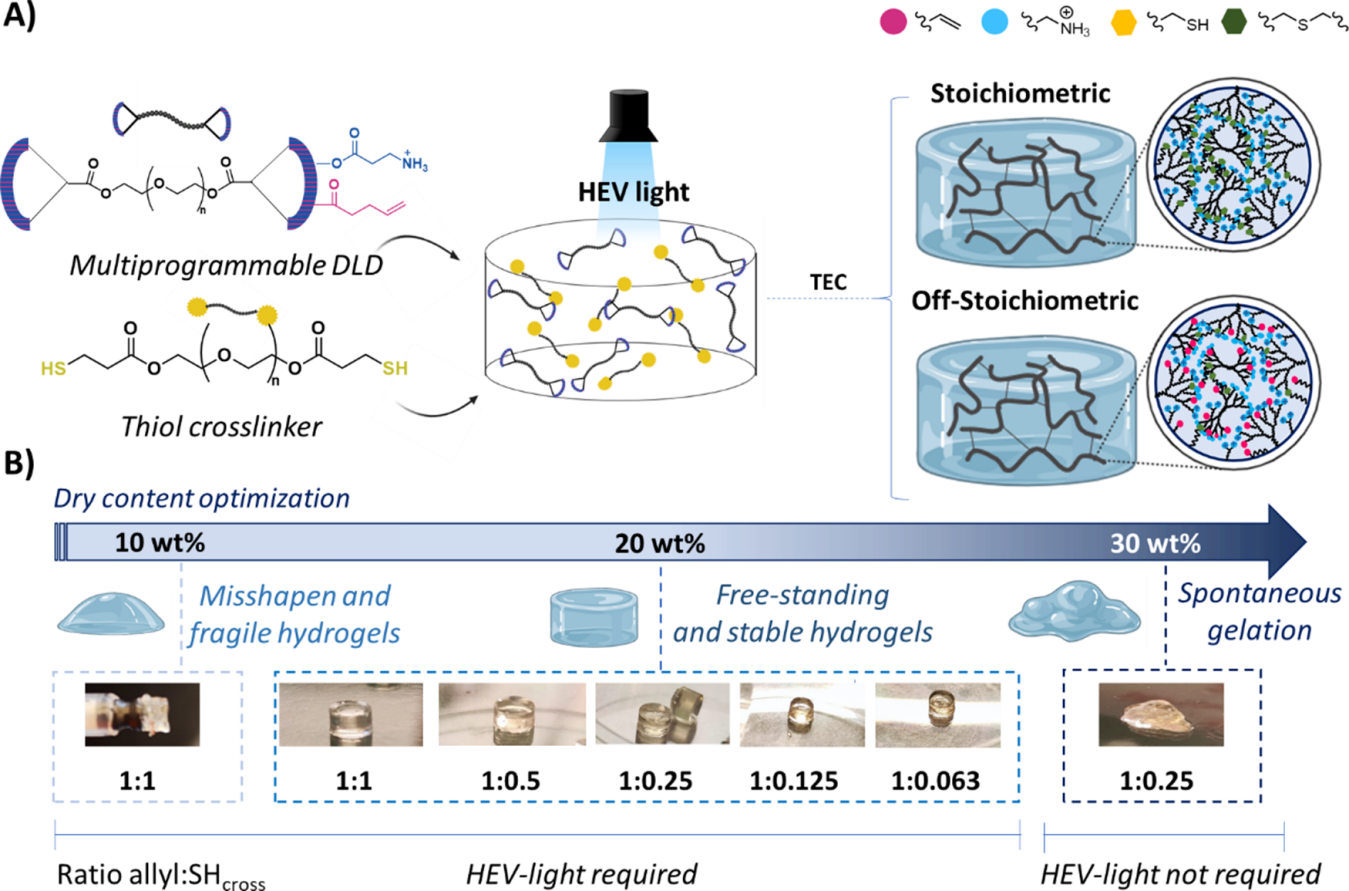
(A) Preparation of ammonium-decorated dendritic hydrogels based on thiol–ene click chemistry (TEC) with two different approaches: stoichiometric and off-stoichiometric ratios between the allyls of the DLDs and the thiols of the cross-linkers. (B) Optimization of dry content (10, 20, and 30 wt %) and molar ratios allyl:SH_cross_ (1:1, 1:0.5, 1:0.25, 1:0.125, and 1:0.063).

The formation of the hydrogels was achieved within seconds in aqueous conditions via HEV-TEC chemistry between the allyl functionalities present on the AB_2_C external groups of the DLDs and the thiol groups located on the PEG-based cross-linker PEG2k-SH, synthesized as previously described.^18^ For the formation of the hydrogels, two different approaches were considered: stoichiometric and off-stoichiometric (Figure 2A). The gels were made at 20 wt % dry content. To distinguish from the nomenclature used to refer to the DLDs themselves, hydrogels are named as H*X*k-G*n* (where *X* = 10 or 20, and *n* = 3, 4, 5) depending on the cationic dendritic polymer present in the network.

When stoichiometric conditions were used, the gels were transparent, free-standing, and with a well-defined shape, indicating high stability. Regarding the off-stoichiometric gels, a wide variety of allyl:SH_cross_ molar ratios were tested: 1:0.5 (50% free pending allyls), 1:0.25 (75% free pending allyls), 1:0.125 (87.5% free pending allyls), and 1:0.063 (93.7% free pending allyls). In all cases, even when just 6.3% of the allyls were cross-linked, free-standing and yellowish transparent hydrogels were obtained, the stickiness of which increased with decreasing molar ratio (Figure 2B). The light-yellow color presented by the hydrogels could be due to the presence of free double bounds in the structure of the network. Altering the dry content significantly impacted the consistency of the hydrogels. When the dry content was reduced from 20 to 10 wt %, the resulting hydrogels showed high fragility and lost their shape, even at a allyl:SH_cross_ ratio of 1:1. Increasing the dry content to 30 wt %, resulted in spontaneous gelation without HEV light, even at a low allyl:SH_cross_ ratio of 1:0.25 (Figure 2B). Therefore, 20 wt % was chosen as the optimal dry content for further evaluations.

### Antibacterial Activity of Cationic DLDs

**Figure 3:**
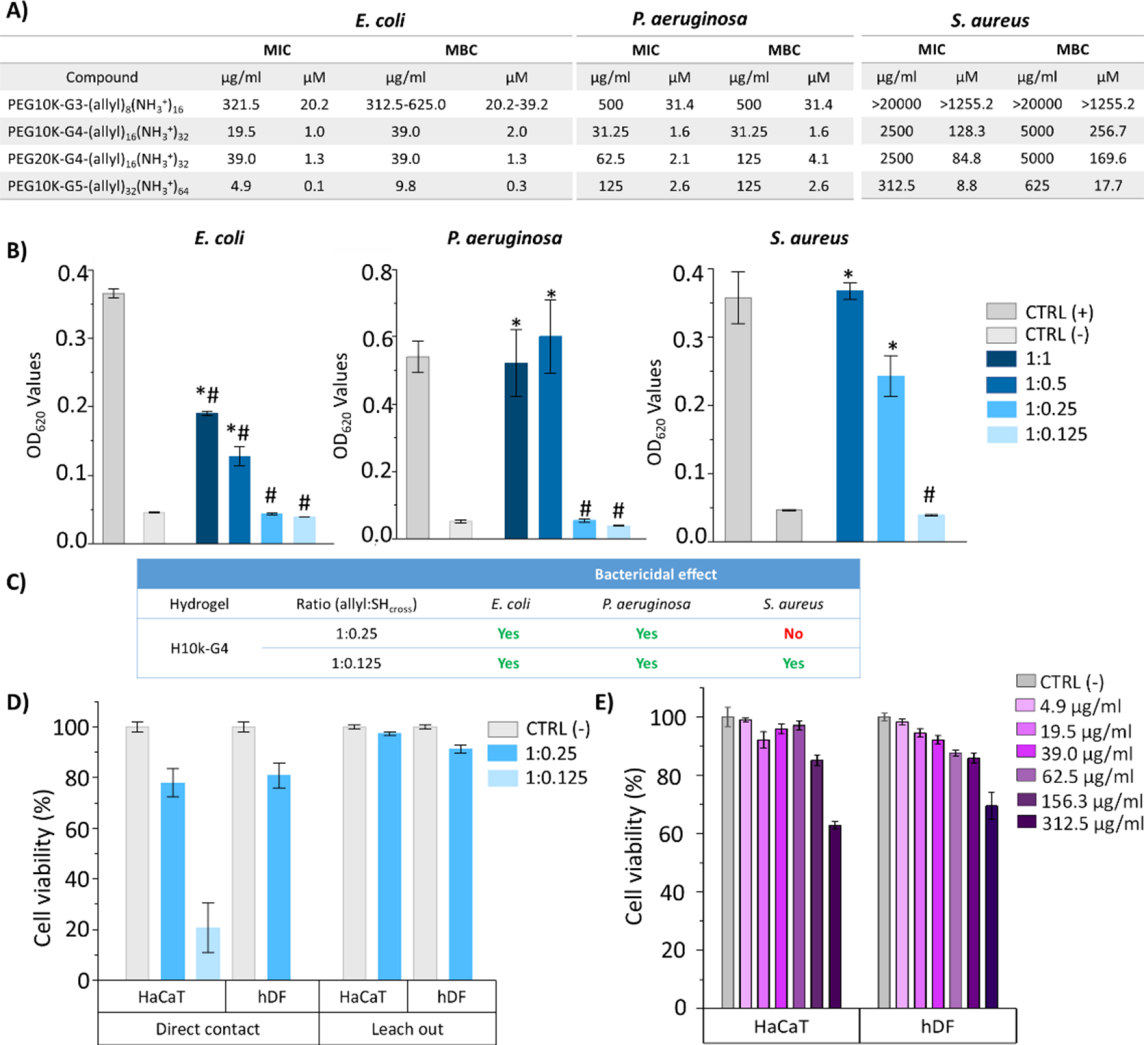
Antibacterial activity evaluation toward Gram-negative (*E. coli* 178 and *P. aeruginosa* 22.644) and Gram-positive (*S. aureus* 2569) planktonic bacteria strains. (A) Minimal inhibitory concentration (MIC) and minimal bactericidal concentration (MBC) for the new family of multiprogrammable DLDs functionalized with allyl moieties and ammonium groups. (B) Antibacterial activity of hydrogels H10k-G4 at different molar ratios in bacterial solution at a concentration of 10^4^ compared to untreated bacteria solution as a positive control (CTRL (+)) and culture medium as a negative control (CTRL (−)). (C) Bactericidal effect of hydrogels. (D) Toxicity of hydrogels toward human keratinocyte cells (HaCaT) and human dermal fibroblasts (hDF) by direct contact and leach out experiments. (E) Toxicity of the polymer PEG10k-G4-(allyl)_16_(NH_3_^+^)_32_ after 24 h of incubation with HaCaT and hDF at different concentrations. Mean values shown with error bars showing standard deviation, *n* = 3. Statistical analysis: One-way ANOVA (Bonferroni). # *p* < 0.5 vs CTRL (+); * *p* < 0.5 vs CTRL (−).

The minimum inhibitory concentration (MIC) and minimum bactericidal concentration (MBC) assays were used to evaluate the antibacterial activity of the amphiphilic AB_2_C functionalized DLDs from the third to the fifth generation toward Gram-negative (*E. coli* 178 and *P. aeruginosa* 22.644) as well as Gram-positive (*S. aureus* 2569) as model bacteria strains. For the fourth-generation derivative, two different PEG chain lengths (10k and 20k) were evaluated in order to analyze the effect of the PEG length on the antibacterial performance. The obtained results are summarized in Figure 3A. Generally, an increase in the dendritic generation was accompanied by higher antibacterial activity against *E. coli* and *S. aureus*, with the fifth-generation PEG10k-G5-(allyl)_32_(NH_3_^+^)_64_ showing the highest activity, especially against *E. coli*, with a MIC value of 0.1 μM. This concentration was 200 times lower than that of the equivalent third-generation DLD. However, against *P. aeruginosa*, the PEG10k-G4-(allyl)_16_(NH_3_^+^)_32_ DLD displayed the most promising activity, with a MIC value 1.6 times lower than that of the equivalent fifth generation, despite it having half the number of cationic groups. In spite of the well-known higher resistance offered by Gram-negative bacteria compared to Gram-positive bacteria,^26^ all cationic DLDs showed stronger activity against Gram-negative bacteria. The PEG10k-G5-(allyl)_32_(NH_3_^+^)_64_ DLD was the only one that presented activity toward *S. aureus* but with a MIC concentration 88 times higher than in *E. coli*. In contrast with the dendritic generation effect, the length of the PEG chain did not seem to have an appreciable effect on the antibacterial activity of the DLDs.

The antibacterial activity of the noncharged dendritic polymers PEG*X*k-G*n*-(allyl)_*m*_(OH)_2*m*_ from the third to the fifth generation was evaluated toward *E. coli* and *S. aureus* (Figure S9). In order to evaluate the influence of the presence of the allyl functionalities on the antibacterial performance, homofunctionalized PEG10k-G3-(NH_3_^+^)_16_ and PEG20k-G4-(NH_3_^+^)_32_ DLDs were also evaluated against *E. coli* (Figure S9). The lack of activity shown by the hydroxyl-functionalized precursors (PEG*X*k-G*n*-(allyl)_*m*_(OH)_2*m*_), with MIC/MBC values higher than 5 mg/mL, confirmed that the positive charges were the main providers of antibacterial activity in the PEG*X*k-G*n*-(allyl)_*m*_(NH_3_^+^)_2*m*_ DLDs. Interestingly, when comparing the antibacterial activity of the cationic homo- and heterofunctionalized DLDs, we observed that, with the same number of positive charges, the presence of the allyl functionalities increased the antibacterial activity by approximately 16 times in *E. coli*. We hypothesize that the presence of the hydrophobic chains with alkene moieties might help to disrupt the bacterial membrane by enhancing their interaction with lipidic bilayers.

### Biological Evaluation of Cationic Hydrogels

The promising antibacterial activity obtained from the cationic DLDs led us to evaluate the antibacterial activity of the hydrogels prepared from the third to the fifth generation PEG*X*k-G*n*-(allyl)_*m*_(NH_3_^+^)_2*m*_ DLDs together with the PEG-2kSH cross-linker. For this experiment, the same bacteria strains as in the MIC/MBC evaluations were used (*E. coli*, *P. aeruginosa*, and *S. aureus*). Low toxicity is crucial for potential antibacterial materials, and therefore the cytotoxicity was evaluated with cell viability measurements according to ISO10993–5:2009.^27^

The screening of the antibacterial activity in the solution showed that both the allyl:SH_cross_ molar ratio used in the preparation of the hydrogel as well as the generation of the dendritic polymer had a notable effect on the antibacterial properties of the hydrogels (Figure S11A). None of the stoichiometric hydrogels showed significant antibacterial activity toward any of the tested bacteria. This lack of activity was likely due to the inaccessibility of the ammonium groups caused by the high cross-linking density of the hydrogel. This explanation was corroborated after the evaluation of the antibacterial activity of the off-stoichiometric hydrogels, where a reduction in the cross-linking density was associated with an increase in the antibacterial activity against both Gram-positive and Gram-negative bacteria.

Similarly to what was previously observed for the DLDs, the hydrogel formulations prepared from the third-generation derivative, with the lowest number of ammonium groups, were the ones with the lowest antibacterial activity, showing a bacteriostatic effect with a molar ratio of 1:0.25 (allyl:SH_cross_), but no bactericidal effects against any of the tested bacteria strains (Figure S11B). There was not a clear relationship between dendritic generation and antibacterial activity for the hydrogels containing the fourth and fifth-generation DLDs. The H10k-G4 hydrogel prepared with a ratio of 1:0.25 was the most promising, as it killed 100% of all Gram-negative bacteria (*E. coli* and *P. aeruginosa*) (Figure 3C) and was not toxic toward human keratinocytes (HaCaT cells) and human dermal fibroblasts (hDF) through direct contact and leach out experiments (Figure 3D). Additionally, disk diffusion experiments confirmed that this hydrogel also presented activity against both *E. coli* and *P. aeruginosa* by direct contact, with inhibition zone diameters of 0.6 cm in both cases (Figure S13). The outstanding performance of this formulation toward Gram-negative bacteria led us to further investigate the biocompatibility of the dendritic precursor. An analysis of the cell viability of HaCaT and hDF cells after their exposure to different concentrations of PEG10k-G4-(allyl)_16_(NH_3_^+^)_32_ showed high biocompatibility at the concentrations at which this derivative was active toward Gram-negative bacteria (Figure 3E).

Surprisingly, the antibacterial effect toward *S. aureus* was only achieved for the same system by decreasing the ratio even further to 1:0.125; however, at this ratio, the hydrogel showed toxicity in HaCaT cells, so it was discarded from further testing (Figure 3D). In general terms, these hydrogels showed narrow-spectrum antibacterial activity with a preference for Gram-negative bacteria. This *in vitro* selectivity is attractive as it could avoid collateral damage to the host microbiome during treatment and may avoid the cross-resistance of nontargeted pathogens.^28^

Interestingly, doubling the number of charges in the DLD from the fourth to the fifth generation did not improve the antibacterial activity of the hydrogels. This might be explained considering the number of ammonium groups present in the volume of the tested hydrogel (Figure S11B), where the fourth-generation derivative with PEG10k had a slightly higher amount (1.4 μmol per 50 μL) than the fifth-generation derivative (1.2 μmol per 50 μL). Additionally, the fifth-generation DLD has twice the number of allyl groups as the fourth-generation counterpart, so even at the same allyl:SH_cross_ ratio, the cross-link density is higher, and therefore the cationic charges would be less accessible.

Finally, in this first screening of the hydrogels, the PEG length appeared to affect the antibacterial activity, in contrast to what was observed for the polymers. The results with PEG10k for the fourth-generation derivative were better than those obtained from the equivalent PEG20k derivative, at the ratio 1:0.25 allyl:SH_cross_, where the latter showed a bacteriostatic effect toward Gram-negative bacteria both in solution and by direct contact (inhibition zone of 0.7 cm in both cases, Figure S13), but only showed a bactericidal effect toward *E. coli* and not *P. aeruginosa* (Figure S11). This appeared to be due to the difference in the number of ammonium groups per volume of hydrogel, which was lower for the H20k-G4 hydrogel than the H10k-G4 hydrogel. Collectively, all these results pointed to the formulation H10k-G4 in a ratio of 1:0.25 as the most promising candidate.

### Adhesion to Different Surfaces

**Figure 4:**
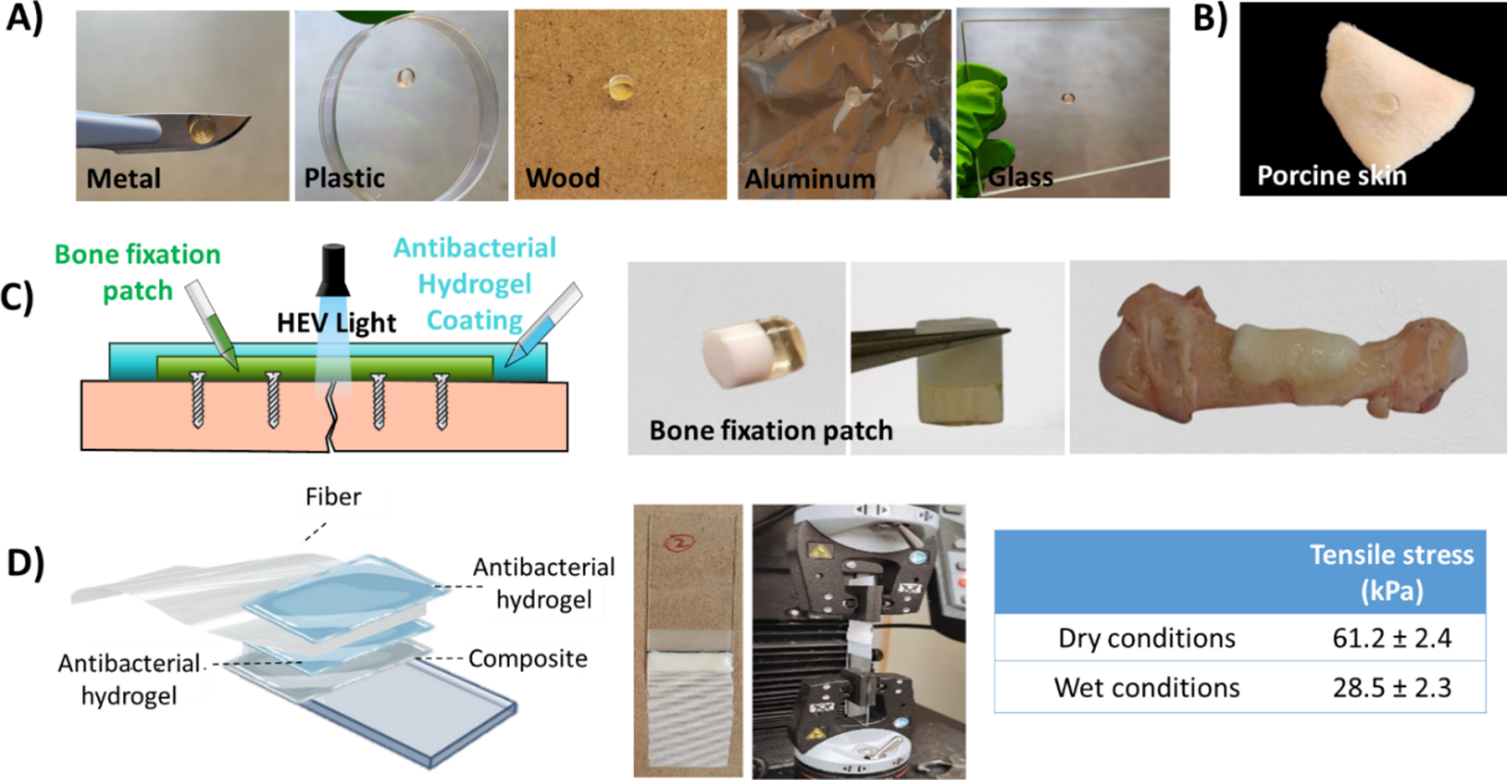
Adhesive properties of ammonium-decorated hydrogels to (A) nonbiological surfaces (metal, plastic, wood, aluminum foil, and glass), (B) biological tissues (pig skin) and (C) composites for bone fixation. (D) Method development to quantify the adhesion of the hydrogel H10KG4 (ratio 1:0.25) to the composite through a peel test. Wet conditions were measured after swelling of the gels overnight in PBS at 37 °C. Results are presented as mean ± SD (*n* = 5).

To obtain good performance in biomedical devices and as coatings in postsurgical infections, strong adhesion between the hydrogels and surfaces is highly desired. The hydrogels were adhered to various surfaces and tissues that can be classified as either nonbiological surfaces (metal, plastic, wood, aluminum, and glass, Figure 4A) or biological tissues (porcine skin, Figure 4B). The hydrogel was also adhered to a hydroxyapatite/polymer composite, which is being developed as a customizable implant for bone fracture fixation (Figure 4C).^24,29^ Adhesion to nonbiological surfaces is desired when targeting antibacterial coatings for medical devices, while adhesion to skin tissues is desirable for topologically applied antibacterial gels. It has been previously reported that the presence of ammonium groups in the structure of the hydrogel can replace hydrated cations and absorb water from the tissue surface, letting the hydrophobic functionalities interact with some skin receptors (thiols, amides, etc.).^30^ Adhesion to the hydroxyapatite/polymer composite is desired, as this would allow for the hydrogel to act as a barrier against surgical site infections during bone fixation surgery. The composite has been previously reported by our research group as being a strong, surgically viable, and nondegradable fixation method for bone fractures, which is based on TEC chemistry. The composite overcomes the soft-tissue adhesion and limited customizability issues of standard-of-care metal fixation implants.^29^

The antibacterial hydrogels showed strong adhesion to all surfaces before and after swelling and were easily applied as a coating on top of a fixation patch on a porcine metacarpal bone, showing good adhesion to the patch after curing with HEV-TEC light (Figure 4C). The presence of an antibacterial coating in bone fixation patches would reduce postsurgical complications and, therefore, the need for antibiotic administration which risks increasing the AMR offered by some bacteria strains and currently carries a large socioeconomic cost.

Moreover, the adhesion was quantified for the most promising hydrogel in terms of antibacterial performance (H10KG4 ratio 1:0.25) toward the composite patch surface using a specially developed method (Figure 4D). A typical lap shear test, with the hydrogel sandwiched between two substrates, could not be used as exposure to HEV light was required to cure the hydrogel. Therefore, the hydrogel was cured on top of the surface of the fixation patch composite with a fiber mesh embedded within the hydrogel. The composite and mesh were pulled apart until failure of the hydrogel/composite construct. Samples were evaluated in dry conditions (after curing) and in wet conditions (after overnight incubation in PBS at 37 °C). The maximum tensile stress value was 61.2 ± 2.4 kPa under dry conditions and 28.5 ± 2.3 kPa under wet conditions. In all cases, the construct failed due to cohesive failure within the hydrogel as opposed to adhesive failure; therefore, the adhesive strength of the hydrogel must exceed these stress values. These stress values exceed those reported in the literature for hydrogels that are described as highly adhesive, where values of 40 kPa have been reported under dry conditions^30^ and 5–7 kPa under wet conditions.^31^

### Three-Component Hydrogels with Biologically Interesting Molecules

**Figure 5:**
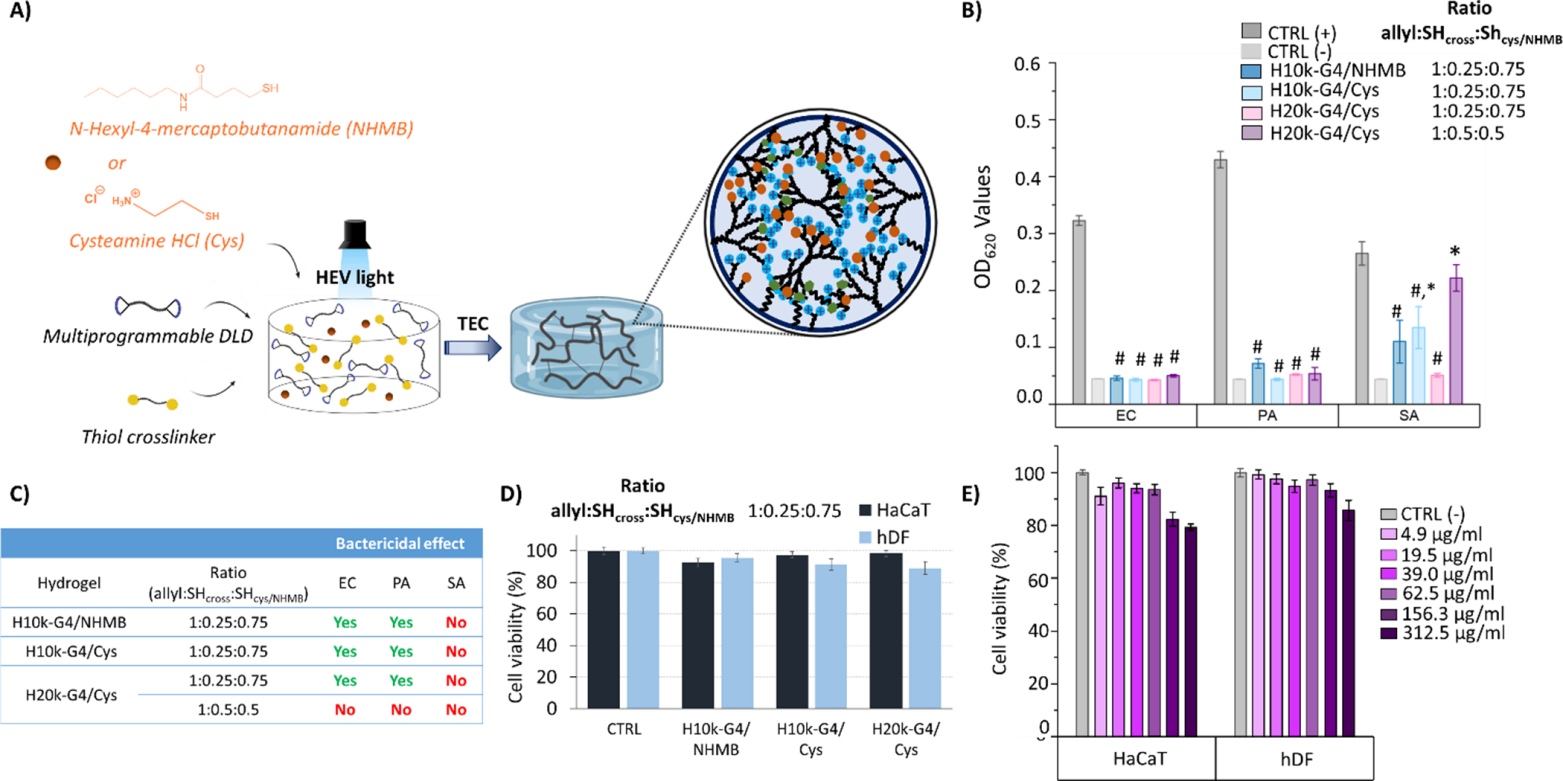
(A) New three-component formulation hydrogels with cysteamine hydrochloride (Cys) and N-Hexyl-4-mercaptobutanamide (NHMB) as additives. (B) Antibacterial activity of hydrogels in bacterial solutions at a concentration of 10^4^ compared to untreated bacteria solution as a positive control (CTRL (+)) and culture medium as a negative control (CTRL (−)). EC: *E. coli* 178, PA: *P. aeruginosa* 22.644, and SA: *S. aureus* 2569. (C) Bactericidal effect of hydrogels. (D) Toxicity of hydrogels toward human keratinocyte cells (HaCaT) and human dermal fibroblasts (hDF) by leach out experiments. (E) Toxicity of the polymer PEG20k-G4-(allyl)_16_(NH_3_^+^)_32_ after 24 h of incubation with HaCaT and hDF at different concentrations. Mean values shown with error bars showing standard deviation, *n* = 3. Statistical analysis: one-way ANOVA (Bonferroni). # *p* < 0.5 vs CTRL (+); * *p* < 0.5 vs CTRL (−).

The presence of unreacted allyls in the off-stoichiometric formulations allowed for the production of three-component hydrogels. To explore this concept, hydrogels were made with either hydrophilic cysteamine hydrochloride (Cys) or N-hexyl-4-mercaptobutanamide (NHMB; Figure 5A). Cys was of interest as it represents a source of positive charges in addition to the β-alanine on the DLDs. While the free form of Cys did not show antibacterial activity (Figure S9), Cys has shown excellent activity against both Gram-positive and Gram-negative bacteria when attached to bis-MPA dendrimers.^16^

The inclusion of Cys also resulted in a heterogeneous environment, with some ammonium groups belonging to β-alanine with TFA as a counterion and others contributed by Cys with chloride as a counterion. NHMB was of interest as the MIC/MBC results of the DLDs showed an antibacterial effect from the allyl groups, suggesting that hydrophobic chains may provide an alternative mechanism for increasing antibacterial activity, possibly through improving the interaction with the bacterial membrane. Due to its promising results, the H10k-G4 gel, with an allyl:SH_cross_ ratio of 1:0.25, was chosen as the starting point for the three-component hydrogels. Cys or NHMB were added to the precursor mixture of H10k-G4 and thiol-cross-linker at an allyl:SH_cross_:SH_cys_/_NHMB_ ratio of 1:0.25:0.75, after which HEV-light-induced TEC cross-linking generated the three-component hydrogels in a one-pot reaction. Following this strategy, all allyls were consumed by the thiol groups on the cross-linker and Cys or NHMB (stoichiometric conditions). The limited solubility of NHMB in water meant that 16% DMSO was required to generate a transparent and homogeneous gel. The new H10k-G4/Cys and H10k-G4/NHMB gels were bactericidal toward Gram-negative bacteria and partially inhibited the growth of Gram-positive bacteria but were not able to fully inhibit or kill *S. aureus*, showing similar behavior regardless of the presence of cationic groups or hydrophobic chains (Figure 5B). The effect of adding Cys to the hydrogels was further evaluated by creating three-component hydrogels with the H20k-G4 DLD (H20k-G4/Cys) at ratios of 1:0.25:0.75 and 1:0.5:0.5 (allyl:SH_cross_:SH_cys_).

The new formulations with Cys were also bactericidal toward Gram-negative bacteria (*E. coli* and *P. aeruginosa*). The hydrogels at a allyl:SH_cross_:SH_cys_ ratio of 1:0.25:0.75 also showed inhibition zones toward Gram-negative bacteria in direct contact experiments, with diameters between 0.63 and 0.73 cm (Figure S13). The formulation H20k-G4/Cys at a ratio of 1:0.25:0.75 allyl:SH_cross_:SH_cys_ was the most interesting since it even showed a bacteriostatic effect toward *S. aureus* in solution and could expand the scope of the two-component formulations when targeting infections that require broad-spectrum treatments (Figure 5B,C). The stickiness of these new formulations hindered the evaluation of the cytotoxicity by direct contact; however, leach out experiments showed good biocompatibility with values of cell viability higher than 70% in both HaCaT and hDF cells (Figure 5D). The presence of the PEG20k-G4-(allyl)_16_(NH_3_^+^)_32_ in the most promising three-component formulation led us to also evaluate its biocompatibility *in vitro* toward HaCaT and hDF cells. The DLD did not show toxicity at the tested concentrations, including the ones in which it showed activity toward Gram-negative bacteria (Figure 5E).

### Evaluation of Mechanical Properties

**Figure 6:**
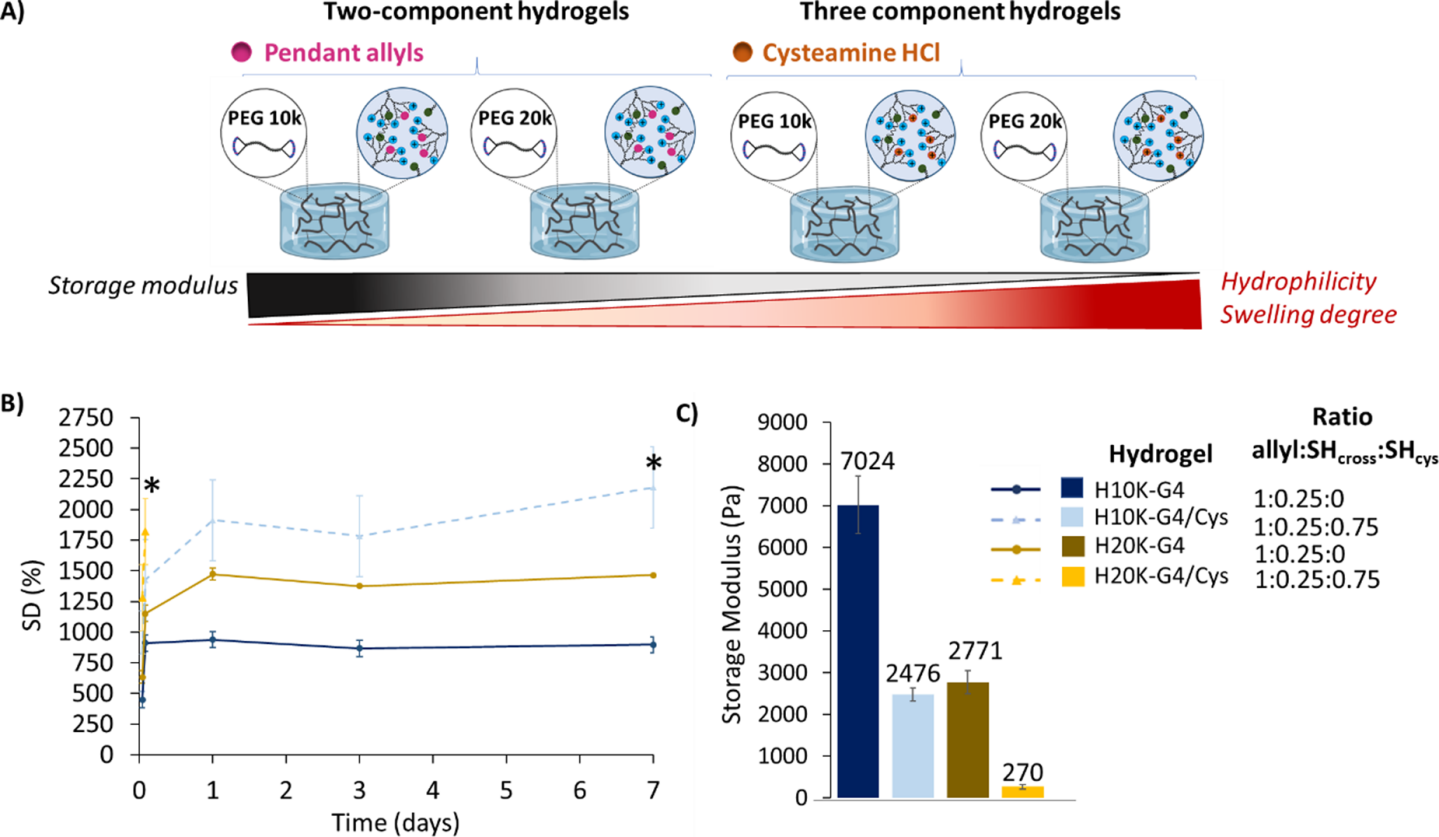
Mechanical properties evaluation for the highly tunable hydrogels based on the fourth-generation DLD with and without Cys. (A) Representation of all tested hydrogels. (B) Swelling degree (SD) as a function of swelling time up to 7 days for hydrogels swollen in PBS at 37 °C. The star (*) represents when the gel has fully or partially degraded. (C) Storage modulus for hydrogels after overnight swelling in PBS at 37 °C. Mean values shown with error bars showing standard deviation, *n* = 5 (swelling experiments) and *n* = 4 (storage modulus measurements).

The hydrogel curing and the mechanical properties of the most promising candidates (H10k-G4 and H20k-G4 with and without Cys) were analyzed by rheology (Figures 6A–C, S16, and S17). As is reported in the literature, the presence of ammonium groups in the systems hinders their characterization by Raman spectroscopy due to the intrinsic fluorescence of those functionalities.^32^ However, by using a rheometer coupled with a UV-curing system and monitoring the increase in storage modulus upon curing, we could determine the time required to achieve fully cured hydrogels. For these experiments, the hydrogel precursor solution was placed in the rheometer and premixed for 2 s before the measurement. For all formulations, less than 20 s was required for the storage modulus to reach a stable plateau (Figure S17). The swelling degree (SD) was also evaluated and related to the storage modulus of these systems after 16 h incubated in PBS at 37 °C (Figure 6). The higher hydrophilicity achieved by the presence of Cys and the use of the longer PEG20k as opposed to PEG10k led to a higher swelling capacity (Figures 6B and S16) and a lower storage modulus (Figure 6C). These small changes to the DLD structure and charges resulted in significant variations to the mechanical properties of the hydrogels, demonstrating the high tunability of this hydrogel platform.

Comparing the extremes of the study, the formulation H10k-G4 with pendant allyls (ratio 1:0.25 allyl:SH_cross_) remained stable up to 56 days (Figure S16) with a storage modulus of 7024 Pa and the lowest SD, whereas the H20k-G4/Cys (ratio 1:0.25:0.75 allyl:SH_cross_:SH_cys_) formulation, with a longer PEG chain and additional Cys groups, displayed a storage modulus 26 times lower, at 270 Pa, an increased SD and the lowest stability: after 3 h, no more swelling data could be obtained due to the high degradability of the system. This tunability allows for the development of antibacterial hydrogels with controlled degradation profiles, swelling capacities, and storage moduli. The versatility offered, particularly in the context of storage modulus, is highly desired when targeting biomedical applications due to the large variability of modulus depending on the body tissue, which ranges from 0.1 to 1 kPa for soft brain tissue to 24–40 kPa for cross-linked osteoid collagen.^33^

## Conclusions

A new generation of telechelic dendritic polymers with orthogonal functionalities from the second to the fifth dendritic generation has been successfully synthesized utilizing various PEG lengths. The presence of both ammonium and allyl groups resulted in DLDs with excellent antibacterial activity against planktonic Gram-positive and Gram-negative bacteria, which improved with increasing dendritic generation. The use of these systems as hydrogel components gave rise to highly versatile networks that could be cured on demand within seconds in aqueous conditions through stoichiometric and off-stoichiometric approaches. A decrease in the cross-linking density in the network provided higher antibacterial activity, especially toward Gram-negative bacteria strains. The hydrogel H10k-G4 in an allyl:SH_cross_ ratio of 1:0.25 displayed the most promising antibacterial activity and low toxicity toward HaCaT and hDF as skin model cells. This material also showed strong adhesion to nonbiological surfaces, biological tissues, and bone fixation patches.

A new method developed by the group allowed the quantification of the adhesion in dry and wet conditions of the hydrogel to a composite surface, showing promising adhesion values even after swelling overnight. Additionally, the presence of allyl functionalities in the off-stoichiometric networks opened up many possibilities of functionalization with both hydrophilic and hydrophobic biologically interesting molecules such as Cys or NHMB. Remarkably, the hydrogel H20k-G4/Cys in a molar allyl:SH_cross_:SH_cys_ ratio of 1:0.25:0.75 showed broader antibacterial activity than that observed for the two-component hydrogels, including a bacteriostatic effect toward *S. aureus* while maintaining good biocompatibility, but more interestingly, its inclusion drastically changed the mechanical properties of these networks. The inclusion of Cys also reduced the storage modulus and resulted in much faster degradability promoted by a high water uptake due to the presence of more hydrophilic functionalities throughout the structure of the network. These results show that with a careful selection of the biomolecule included as an additive, desired properties such as degradability, swelling capacity, or storage modulus can be tuned. The high versatility of this new generation of antibacterial materials, together with their adhesion to surfaces and good biocompatibility, place them as interesting alternatives to conventional antibiotics.

## Supporting Information Available

Detailed methods and synthetic protocols together with spectroscopy data (^1^H NMR, ^13^C NMR, and SEC); screening of antibacterial activity of hydrogels; captured images of human keratinocytes (HaCaT) and human dermal fibroblasts (hDF) in leach out experiments; and swelling and rheological measurements (PDF)

## Notes

The raw data used to calculate the results in this manuscript are available in the following public repository: 10.5281/zenodo.11442132.
