# Supplementary material for: Antibacterial Hydrogel Adhesives Based on Bifunctional Telechelic Dendritic-Linear–Dendritic Block Copolymers
Source: J Am Chem Soc. 2024 Jun 12;146(25):17240–9. doi: 10.1021/jacs.4c03673 (PMC11212050; doi:10.1021/jacs.4c03673)
Supplement: Supplementary file 1 — ja4c03673_si_001.pdf [file ja4c03673_si_001.pdf]

Electronic Supporting Information

Antibacterial Hydrogel Adhesives based on Bifunctional Telechelic Dendritic-Linear-Dendritic Block Copolymers

Natalia Sanz del Olmo, Noemi Molina, Yanmiao Fan, Faridah Namata, Daniel J. Hutchinson, Michael Malkoch*.

Department of Fibre and Polymer Technology, KTH Royal Institute of Technology, Teknikringen 56-68, 100 44, Stockholm, Sweden.

Corresponding author: Prof. Michael Malkoch

Contact info: School of Engineering Sciences in Chemistry, Biotechnology and Health; Department of Fibre and Polymer Technology; Division of Coating Technology Teknikringen 48, SE-10044, Stockholm

Fax: (+) 46 (0)8 790 82 83

E-mail: malkoch@kth.se

Materials and Methods

Materials. All materials and solvents were purchased from Sigma Aldrich and used as received unless otherwise noted. 2,2-bis(methylol)propionic acid (bis-MPA) was kindly donated by Perstorp AB, Sweden. DOWEX 50WX2 50-100 (H) was purchased from Acros Organics.

Escherichia coli 178 (*E. coli* 178) was kindly provided by Professor Paul Orndorff (North Carolina State University). *Staphylococcus aureus 2569* (*S. aureus* 2569) and *Pseudomonas aeruginosa 22.644* (*P. aeruginosa 22.644*) were purchased from DSMZ. Human epidermal keratinocytes (HaCaT) and Human Dermal Fibroblasts (hDF) were purchased from the American Tissue Culture Collection (ATCC). For the tests of cell viability, Dulbecco's Modified Eagle Medium (DMEM), fetal bovine serum (FBS) and the mixture of antibiotics penicillin/streptomycin were purchased from Thermo Fisher Scientific.

Characterization Methods. *NMR Spectroscopy.* ^{1}H and ^{13}C NMR spectroscopy were performed on a 400 MHz Bruker Avance III. ^{1}H NMR spectroscopy was performed using a 1 s relaxation delay and 20 ppm spectral window. The respective values for ^{13}C NMR spectroscopy were 2 s and 240 ppm. Samples were analyzed in deuterated solvents ($CDCl_3$ and CD_3OD). ^{1}H-NMR spectra were referenced to the residual solvent peak of $CDCl_3$ δ 7.24, CD_3OD δ 3.31, and ^{13}C-NMR spectra to $CDCl_3$ δ 77.0 and CD_3OD δ 49.0. The spectra were analyzed with MestreNova v. 14.2.0-26256 from Mestrelab Research S.L.

Size Exclusion Chromatography (SEC). SEC was performed on a TOSOH EcoSECHLC-8320GPC system equipped with an EcoSES RI detector and three columns from PSS GmbH were used (PSS PFG 5 μm; Microguard, 100 Å and 300 Å). The mobile phase was DMF with 0.01M LiBr (0.2mL min-1) at 50 °C using a conventional calibration method with polyethylene glycol (PEG) standards. Corrections for flow rate fluctuations were made using toluene as an internal standard.

Formulation of cationic hydrogels. The formation of the hydrogel was achieved via High-energy visible light (HEV) initiated thiolene click chemistry (TEC) between the allyl functionalized DLDs based on AB_2C and the thiol groups present in the PEG-based crosslinker (PEG2k-SH) and the thiols present in the additives of the three component hydrogels. Different

allyl to thiol crosslinker ratios of 1:1, 1:0.5, 1:0.25, 1:0.125 and 0.063 were used. Lithium phenyl-2,4,6-trimethylbenzoylphosphinate (LAP) was added as a photo-initiator at a concentration of 2.4 wt %. The contents (DLD, crosslinker, photoinitiator and, cysteamine hydrochloride or N- hexyl-4-mercaptobutanamide (NHMB) for the three-component hydrogels) were dissolved in water at a concentration of 20 wt %. The gels containing the hydrophobic thiol as additive needed 16 % of DMSO. Curing was achieved in a cylindrical mold by a portable high-performance curing LED lamp (Bluephase 20i, Ivoclar Vivadent AG, Leichtenstein) with dominant wavelengths of 400 and 470 nm and an intensity of 1200 $mWcm^{-2}$. LED treatment with 2 pulses (20 s/pulse) was applied resulting in solid and transparent hydrogels.

Swelling. Swelling evaluation was performed by submerging and keeping the hydrogels in PBS buffer (pH = 7.4, phosphate buffer 0.01 M, NaCl 0.154 M) at 37 ^{o}C. The dry weight of the materials (Wd) was obtained by drying the hydrogels in a vacuum oven at 50 °C overnight. The weight of the swollen materials (Ws) was obtained at different time points. The degree of swelling was calculated using Equation 1. Five repeats were performed for each sample, and all results are shown as mean ± SD.

$$Degree\ of\ swelling = \frac{Ws - Wd}{Wd}\ x\ 100 \qquad \text{(Equation 1)}$$

Rheological analysis. Rheological measurements for the hydrogels were carried out on a TA Discovery Hybrid Rheometer (DHR-2) with a plate/plate geometry equipped with a Peltier plate accessory using a stainless-steel upper geometry (Ø = 8 mm). For the amplitude sweep experiments, hydrogels were swollen at 37 ^{o}C for 16 h. After addition of the hydrogel to bottom plate, the upper plate was lowered to a final gap of 600 µm and with a frequency of 1 Hz and oscillation strains ranging from 0.1 %–1000 %. Time sweep experiments were performed at room temperature on the formulations using the UV curing accessory from TA Instruments. Analyses were carried out for 60 s with 15 s of conditioning before start. For all rheological measurements, four repeats were performed for each sample, and all results are shown as mean ± SD.

Antibacterial activity evaluation of the cationic DLDs. Minimum inhibitory concentration (MIC) and minimum bactericidal concentration (MBC) assays were used to evaluate the antibacterial activities of the cationic DLDs towards *E. coli 178*, *S. aureus 2569 and P. aeruginosa 22.644*. For MIC evaluation, samples were diluted with sterilized water using the double dilution method. Bacterial solutions at log phase were diluted with MHB II broth to

reach the concentration of 10^6 CFU mL^{-1}. After inoculation, equal volume (50 μL) of the microorganism was incubated with biocides and controls in sterile 96-well plates at 37 °C for 18 h with shaking of 250 rpm. The final optical density was then measured using the Multiskan FC Microplate reader (Thermo Fisher scientific (Shanghai) instruments Co., Ltd.) using an OD of 620 nm to determine MIC. The negative controls comprise the inoculum, bacteria without biocide, and the culture medium, sample without inoculum and biocide. Amoxicillin was used as a positive control. The MBC was measured by subculturing during 24 h the broths used for MIC determination onto fresh agar plates, concentrations at MIC value and higher were used to check bacterial growth on the fresh agar and determine MBC values. Three repeats were performed for each sample, and all results are shown as mean ± SD.

Cytotoxicity evaluation of the cationic DLDs. A monolayer of human epidermal keratinocytes (HaCaT) and human dermal fibroblasts (hDF) was used for the cytotoxicity tests. These cell lines were maintained in tissue culture flasks at 37 °C in 5 % CO_2 with Dulbecco's Modified Eagle Medium (DMEM), supplemented with 10 % (v/v) Fetal Bovine Serum, L-glutamine (4 mM), 100 IU ml^{-1} penicillin and 100 μg ml^{-1} streptomycin. Cells were harvested and transferred into 96-well plates at a concentration of 5×10^5 cell ml^{-1} in 100 μL of DMEM and cultured for 24 h before use. Afterwards, cells were treated with the polymers dissolved in cell culture at different concentrations (4.9, 19.5, 39.0, 62.5, 156.3 and 312.5 μg/ml). Finally, the treatment was removed and new cell culture containing 10% Alamar Blue was added and incubated for 4 hours at 37 °C in 5 % CO_2. Finally, fluorescent intensity was measured using an Infinite® M200 (Tecan, Switzerland) plate reader at the wavelength of 560/590 nm (excitation/emission). Three repeats were performed for each sample, and all results are shown as mean ± SD.

Antibacterial activity evaluation of the cationic hydrogels. *Solution method. E. coli 178*, *S. aureus 2569* and *P. aeruginosa 22.644* were cultured in MHB II broth at 37 °C, and the bacterial solution at log phase was diluted to the concentration of 10^4 CFU mL^{-1}. The cylindrical hydrogels (50 μL, 20 wt.%) were first swollen in sterilized DI water for 30 min in the fridge, and then added into the 48-well-plate containing 200 μL bacterial solution of 10^4 CFU/mL, to finally be incubated at 37 °C for 24 h. After the incubation, OD values were used to evaluate the bacteriostatic effect of the hydrogels. To determine the bactericidal capacity, 50 μl of solution were deposited to agar plates and incubated at 37 °C overnight. Bacterial solution without hydrogel treatment was used as a positive control and pure medium was use as negative

control. Three repeats were performed for each sample, and all results are shown as mean ± SD.

Disk diffusion method. E. coli 178, *S. aureus 2569 and P. aeruginosa 22.644* were cultured in MHB II broth at 37 °C, and the bacterial solution at log phase was used to prepare the bacterial agar plates (concentration ≈1×10^6 CFU/mL). Hydrogels (50 µL, 20 wt.%) were deposited on bacterial agars and incubated at 37 °C overnight. The diameters of the inhibition zones were measured. Three repeats were performed for each sample, and all results are shown as mean ± SD.

Cytotoxicity evaluation of the cationic hydrogels. A monolayer of human epidermal keratinocytes (HaCaT) and human dermal fibroblasts (hDF) was used for the cytotoxicity tests. These cell lines were maintained in tissue culture flasks at 37 °C in 5 % CO_2 with Dulbecco's Modified Eagle Medium (DMEM), supplemented with 10 % (v/v) Fetal Bovine Serum, L-glutamine (4 mM), 100 IU ml^{-1} penicillin and 100 µg ml^{-1} streptomycin.

Direct contact experiment. Cells were harvested and transferred into 48-well plates at a concentration of 5×10^5 cell mL^{-1} in 500 µL of DMEM and cultured for 24 h before use. The cationic hydrogels (50 µL, 20 wt.%) were exposed to UV light for 20 minutes and swollen in sterilized DI water for 30 min in the fridge. Afterwards, hydrogels were added to the 48-well plates and incubated with the cells (37 °C, 5 % CO_2). After 24h, the hydrogels were removed and Alamar Blue reagent was added at a concentration of 10 % of the final volume and the incubation was continued for 4 h. Finally, the fluorescence intensity was measured at 560/590 nm (excitation/emission) using an Infinite® M200 (Tecan, Switzerland) plate reader. Non-treated controls were maintained throughout the cell viability assay. Three repeats were performed for each sample, and all results are shown as mean ± SD.

Leach out experiment. Cells were harvested and transferred into 96-well plates at a concentration of 5×10^5 cell mL^{-1} in 100 µL of DMEM and cultured for 24 h before use. Meantime, the hydrogels were exposed to UV light for 20 minutes. Afterwards, the materials (50 µL, 20 wt.%) were transferred into 1 ml of complete DMEM and incubated at 37 °C for 24 hours to get the leach-out medium (the testing medium). Afterwards, old cell culture medium was replaced by 100 µl of the testing medium per well and incubated for 72 hours. For each sample medium, six parallel wells were used, and three hydrogels were used for each material's formulation. Cells without treatment were used as a control and extracts without cells were

used as blank of this experiment. Then, 10 μL of Alamar Blue agent was added and incubated for 4 hours at 37 °C in 5 % CO_2. Finally, fluorescent intensity was measured using an Infinite® M200 (Tecan, Switzerland) plate reader at the wavelength of 560/590 nm (excitation/emission). Three repeats were performed for each sample, and all results are shown as mean ± SD.

Adhesion experiments. A thin layer of the bone fixation composite with dimensions 25 x 10 mm (width x length), was applied to a glass substrate and cured with HEV initiated TEC chemistry. A layer of the H10k-G_4 ratio 1:0.25 hydrogel was applied across the whole surface of the composite, before curing with HEV light. A second layer of hydrogel, with an embedded polyethylene terephthalate (PET) fiber mesh, with dimensions 25 x 40 mm (width x length), was then applied and cured. The adhesion of the hydrogel to the composite was either tested immediately (dry conditions), or after the construct had been immersed overnight in PBS buffer (pH 7.4) at 37 °C (wet conditions). The adhesion was evaluated using an Instron 5944 universal testing machine (Instron Korea LLC) with a 500 N load cell and a cross-head speed of 5 mm/min in tensile mode. A preload of 0.5 N and a preload speed of 1 mm/min were used. All measurements were conducted at 20 °C with a relative humidity of 50%. The glass substrate and the fiber mesh were attached to the machine in an axial position and pulled apart until the failure of the hydrogel/composite construct. The construct was then inspected to determine if adhesive or cohesive failure had occurred and the tensile stress at failure was calculated as the maximum load divided by the area of the hydrogel/composite contact. All data were collected using Bluehill software (version 3.72). Five specimens were tested for each condition (dry and wet). Results obtained for each specimen can be found on the **Figure S11**.

Statistical analysis. Statistical significance was assessed using Bonferroni's test for multiple comparisons after one-way analysis of variance (ANOVA). Differences were considered significant if $p < 0.05$. Origin 2020 software was used for the analysis.

Synthetic protocols

AB_2C[1] acid and Boc β-alanine anhydride[2] monomers, as well as PEG2k-SH[3] thiol crosslinker have been synthesized according to previously reported protocols. Additionally, homofunctionalized dendritic-linear dendritic (DLD) precursors functionalized with hydroxyl groups at the periphery[3] as well as the DLD functionalized with protonated amines of fourth

generation (PEG20k-G4-(NH_3^+)$_{32}$)[4] used as control in the experiments of antibacterial activity have been synthesized based on previous protocols.

Synthesis of N- hexyl-4-mercaptobutanamide (NHMB)

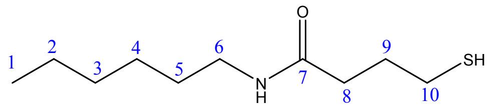

In a RBF equipped with a stirred, hexylamine (9.9 mmol, 1.3 mL) was dissolved in THF and then γ- thiobutyrolactone was added (9.9 mmol, 0.9 mL). The reaction was allowed to proceed overnight at room temperature and was monitored by NMR. The compound was washed with $NaHSO_4$ (10%) three times and obtained as a transparent oil (1.9 g, 95 %). ^{1}H NMR ($CDCl_3$, 400 MHz), δ/ppm: 3.23 (2H, td, J = 7.2, 5.7 Hz, H6), 2.58 (2H, dt, J = 8.0, 7.0 Hz, H10), 2.30 (2H, t, J=7.0, H8), 1.94 (2H, p, J=7.0, H9), 1.54 – 1.40 (2H, m, H5), 1.38 – 1.21 (7H, m, H2, H3, H4 and SH), 0.92 – 0.82 (3H, m, H1). ^{13}C-NMR ($CDCl_3$,101 MHz), δ/ppm: 172.0 (C7), 39.7 (C6), 34.9 (C8), 31.6 (C3), 29.8 (C5) 29.7 (C9), 26.7 (C4), 24.3 (C10), 22.7 (C2), 14.1 (C1).

Synthesis of AB_2C anhydride monomer

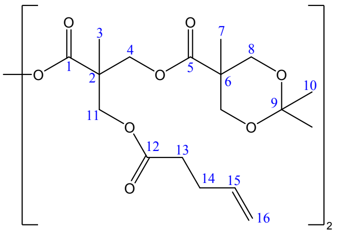

AB_2C anhydride monomer

AB_2C monomer (7.14 g, 0.02 mol) was dissolved in DCM with stirring. The reaction vessel was subsequently cooled in an ice bath. DCC (1.97 g, 0.01 mol) dissolved in DCM was added dropwise over a period of 30 minutes. Once addition was completed, the reaction was allowed to proceed overnight at room temperature. The reaction mixture was filtered and the filtrate was collected and evaporated by rotary evaporation and the product dried *in vacuo* to give **AB_2C anhydride** as a white powder (7.12 g, 98 %). ^{1}H NMR ($CDCl_3$, 400 MHz), δ/ppm: 5.78 (2H, m, H15), 5.03 (4H, m, H16), 4.40-4.24 (8H, m, H4, H11), 4.15 (4H, d, *J*=12 Hz, H8), 3.62 (4H, d, *J*=12 Hz, H8), 2.47-2.31 (8H, m, H13, H14), 1.41 (6H, s, H10), 1.35 (6H, s, H10), 1.33 (6H, s, H3), 1.13 (6H, s, H7). ^{13}C NMR ($CDCl_3$, 101 MHz), δ/ppm: 173.7 (C5), 172.4 (C12), 167.6 (C1), 136.4 (C15), 115.9 (C16), 98.3 (C9), 66.1 (C4, C8, C11), 64.8 (C4, C8, C11), 64.7

(C4, C8, C11), 48.3 (C6), 42.3 (C2), 33.3 (C13), 28.8 (C14), 25.1 (C10), 21.8 (C10), 18.5 (C3, C7), 17.3 (C3, C7).

Synthesis of heterofunctionalized dendritic-linear-dendritic (DLD) polymers

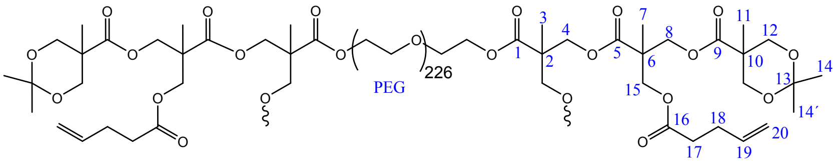

PEG10k-G2-(allyl)$_4$(ac)$_4$

PEG10k-G2-(allyl)$_4$(ac)$_4$. In a RBF equipped with a magnetic stirrer **PEG10k-G1-(OH)$_4$** (5.26 g, 0.5 mmol) was dissolved in DCM. Pyridine (0.5 ml, 6 mmol) and DMAP (73.3 mg, 0.6 mmol) were then added. Afterwards, AB_2C anhydride monomer (2.9 g, 4 mmol) was added to the reaction mixture under vigorous stirring. The reaction was allowed to proceed overnight and monitored by NMR as well as MALDI-TOF-MS. Upon completion the crude reaction was precipitated three times in cold ether and the product was isolated by filtration to obtain **PEG10k-G2-(allyl)$_4$(ac)$_4$** as a white powder (4.7 g, 81 %). ^{1}H NMR ($CDCl_3$, 400 MHz), δ/ppm: 5.80-4.89 (4H, m, H19), 5.10-4.89 (8H, m, H20), 4.38-4.05 (40H, m, H4, H8, H12, H15), 3.63 (910H, s, PEG), 2.46-2.26 (16H, m, H17, H18), 1.40 (12H, s, H14), 1.34 (12H, s, H14), 1.25 (6H, s, H3), 1.23 (12H, s, H7), 1.13 (12H, s, H11). ^{13}C NMR ($CDCl_3$, 101 MHz), δ/ppm: 173.6 (C1, C5, C9, C16), 172.3 (C1, C5, C9, C16), 172.1 (C1, C5, C9, C16), 171.9 (C1, C5, C9, C16), 136.5 (C19), 115.7 (C20), 98.1 (C13), 70.6 (PEG), 68.8 (PEG), 68.3 (PEG), 66.0-63.7 (C4, C8, C12, C15), 46.7 (C6, C10), 46.6 (C6, C10), 42.1 (C2), 33.2 (C17), 28.7 (C18), 25.2 (C14), 22.1 (C14), 18.5 (C3, C7, C11), 17.8 (C3, C7, C11), 17.6 (C3, C7, C11). SEC (DMF) M_n =15030 g mol^{-1}, M_w= 15952 g mol^{-1}, Đ = 1.06.

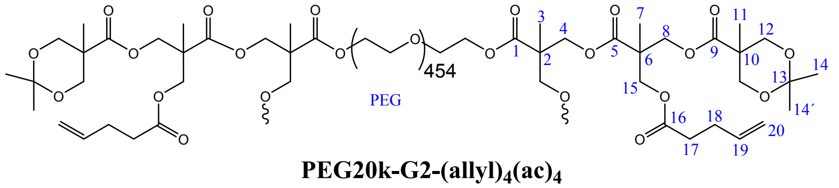

PEG20k-G2-(allyl)$_4$(ac)$_4$

PEG20k-G2-(allyl)$_4$(ac)$_4$. In a RBF equipped with a magnetic stirrer **PEG20k-G1-(OH)$_4$** (2.1 g, 0.1 mmol) was dissolved in DCM. Pyridine (0.2 ml, 2.4 mmol) and DMAP (9.8 mg, 0.08 mmol) was then added. Afterwards, AB_2C anhydride monomer (581.4 mg, 0.8 mmol) was added to the reaction mixture under vigorous stirring. The reaction was allowed to proceed overnight and monitored by NMR as well as MALDI-TOF-MS. Upon completion the crude reaction was precipitated three times in ether and the product was isolated by filtration to obtain

PEG20k-G2-(allyl)$_4$(ac)$_4$ as a white powder (1.9 g, 87 %). ^{1}H-NMR (400MHz, $CDCl_3$), δ/ppm: 5.84-5.70 (4H, m, H19), 5.10-4.92 (8H, m, H20), 4.35-4.04 (40H, m, H4, H8, H12, H15), 3.62 (1818H, s, PEG), 2.45-2.27 (16H, m, H17, H18), 1.39 (12H, s, H14), 1.33 (12H, s, H14), 1.25 (6H, s, H3), 1.23 (12H, s, H7), 1.12 (12H, s, H11). ^{13}C-NMR (101 MHz, CDCl3), δ/ppm: 173.6 (C1, C5, C9, C16), 172.3 (C1, C5, C9, C16), 172.0 (C1, C5, C9, C16), 171.9 (C1, C5, C9, C16), 136.4 (C19), 115.7 (C20), 98.1 (C13), 70.6 (PEG), 68.8 (PEG), 68.3 (PEG), 66.0-63.7 (C4, C8, C12, C15), 46.6 (C6, C10), 46.5 (C6, C10), 42.0 (C2), 33.2 (C17), 28.7 (C18), 25.2 (C14), 22.1 (C14′), 18.5 (C3, C7, C11), 17.7 (C3, C7, C11), 17.6 (C3, C7, C11). SEC (DMF) M_n =27268 g mol^{-1}, M_w= 28687 g mol^{-1}, Đ = 1.05.

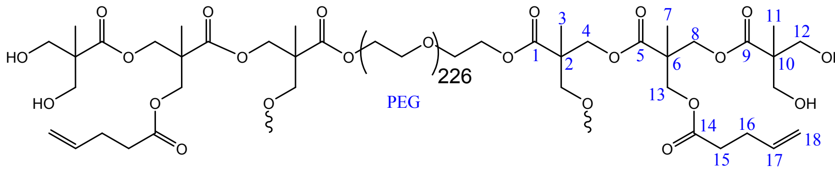

PEG10k-G2-(allyl)$_4$(OH)$_8$

PEG10k-G2-(allyl)$_4$(OH)$_8$. Dowex™ (3.3 g) was added to a solution of **PEG10k-G2-(allyl)$_4$(ac)$_4$** (3.26 g, 0.3 mmol) in DCM/MeOH mixture (10 mL). The reaction was allowed to proceed with stirring at room temperature. Progress was monitored with ^{1}H NMR spectroscopy. After completion, the Dowex™ was removed by filtration and the filtrate was evaporated. The resulting product was dissolved in DCM, precipitated in ether and dried *in vacuo* to give **PEG10k-G2-(allyl)$_4$(OH)$_8$** as a white powder (2.65 g, 77 %). ^{1}H NMR ($CDCl_3$, 400 MHz), δ/ppm: 5.75-5.61 (4H, m, H17), 4.99-4.84 (8H, m, H18), 4.21-4.07 (24H, m, H4, H8, H13), 3.76-3.31 (926H, s, PEG, H12), 2.37-2.17 (16H, m, H15, H16), 1.16-1.14 (18H, s, H3, H7), 0.97 (12H, s, H11). ^{13}C NMR ($CDCl_3$, 101 MHz), δ/ppm: 174.7-172.0 (C1, C5, C9, C14), 136.2 (C17), 115.5 (C18), 70.3 (PEG), 68.6 (PEG), 66.1-64.2 (C4, C8, C12, C13), 49.6 (C10), 46.4 (C6), 33.0 (C15), 28.5 (C16), 17.5 (C3, C7, C11), 17.4 (C3, C7, C11), 17.0 (C3, C7, C11). SEC (DMF) M_n =13526 g mol^{-1}, M_w= 13811 g mol^{-1}, Đ = 1.02.

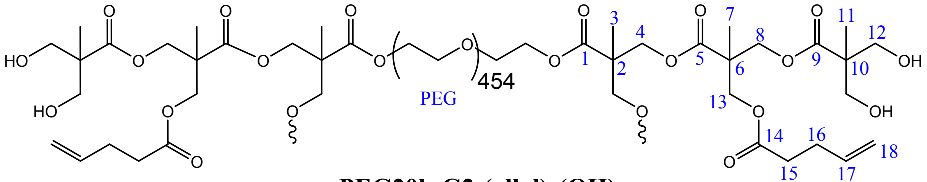

PEG20k-G2-(allyl)$_4$(OH)$_8$

PEG20k-G2-(allyl)$_4$(OH)$_8$. Dowex™ (3.2 g) was added to a solution of **PEG20k-G2-(allyl)$_4$(ac)$_4$** (3.15 g, 0.15 mmol) in DCM/MeOH mixture (10 mL). The reaction was allowed to proceed with stirring at room temperature. Progress was monitored with ^{1}H NMR

spectroscopy. After completion, the Dowex™ was removed by filtration and the filtrate was evaporated. The resulting product was dissolved in DCM, precipitated in ether and dried *in vacuo* to give **PEG20k-G2-(allyl)$_4$(OH)$_8$** as a white powder (2.41 g, 75 %). ^{1}H NMR ($CDCl_3$, 400 MHz), δ/ppm: 5.88-5.71 (4H, m, H17), 5.10-4.96 (8H, m, H18), 4.35-4.16 (24H, m, H4, H8, H13), 3.85-3.41 (1834H, s, PEG, H12), 2.49-2.29 (16H, m, H15, H16), 1.27-1.25 (18H, s, H3, H7), 1.07 (12H, s, H11). ^{13}C NMR ($CDCl_3$, 101 MHz), δ/ppm: 174.9-172.6 (C1, C5, C9, C14), 136.4 (C17), 115.7 (C18), 70.6 (PEG), 68.3 (PEG), 67.3-63.7 (C4, C8, C12, C13), 49.8 (C10), 46.7 (C6), 33.2 (C15), 28.6 (C16), 17.7 (C3, C7, C11), 17.2 (C3, C7, C11), 16.6 (C3, C7, C11). SEC (DMF) M_n = 24291 g mol^{-1}, M_w= 24995 g mol^{-1}, Đ = 1.03.

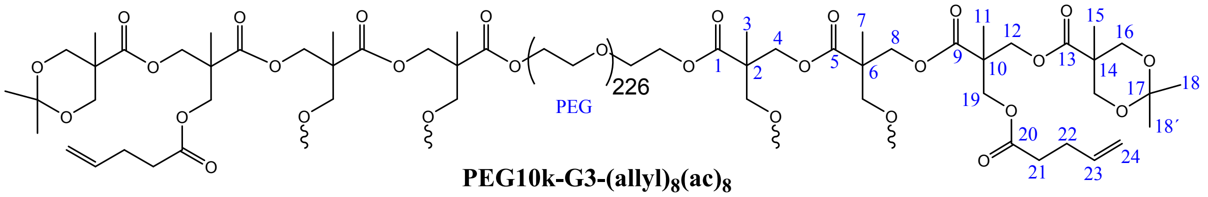

PEG10k-G3-(allyl)$_8$(ac)$_8$

PEG10k-G3-(ally)$_8$(ac)$_8$. In a RBF equipped with a magnetic stirrer **PEG10k-G2-(OH)$_8$** (6.1 g, 0.6 mmol) was dissolved in DCM. Pyridine (1.1 ml, 14.4 mmol) and DMAP (171 mg, 1.4 mmol) were then added. Afterwards, AB_2C anhydride monomer (7.0 g, 9.6 mmol) was added to the reaction mixture under vigorous stirring. The reaction was allowed to proceed overnight and monitored by NMR as well as MALDI-TOF-MS. Upon completion the crude reaction was precipitated three times in ether and the product was isolated by filtration to obtain **PEG10k-G3-(ally)$_8$(ac)$_8$** as a white powder (6.3 g, 78 %). ^{1}H NMR ($CDCl_3$, 400 MHz), δ/ppm: 5.87-5.71 (8H, m, H23), 5.09-4.94 (16H, m, H24), 4.36-4.07 (88H, m, H4, H8, H12, H16, H19), 3.64 (910H, s, PEG), 2.50-2.28 (32H, m, H21, H22), 1.41 (24H, s, H18), 1.35 (24H, s, H18´), 1.24 (42H, m, H3, H7, H11), 1.14 (24H, s, H15). ^{13}C NMR ($CDCl_3$, 101 MHz), δ/ppm: 173.5-171.4 (C1, C5, C9, C13, C20), 136.5 (C23), 115.7 (C24), 98.1 (C17), 70.5 (PEG), 68.7 (PEG), 68.3 (PEG), 66.0-63.6 (C4, C8, C12, C16, C19), 46.7-46.5 (C6, C10, C14), 42.0 (C2), 33.2 (C21), 28.7 (C22), 25.2 (C18), 22.1 (C18), 18.5-17.5 (C3, C7, C11, C15). SEC (DMF) M_n =16504 g mol^{-1}, M_w= 17640 g mol^{-1}, Đ = 1.07.

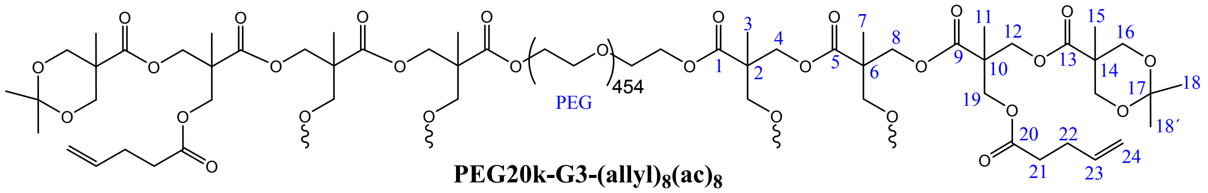

PEG20k-G3-(allyl)$_8$(ac)$_8$

PEG20k-G3-(ally)$_8$(ac)$_8$. In a RBF equipped with a magnetic stirrer **PEG20k-G2-(OH)$_8$** (5.4 g, 0.26 mmol) was dissolved in DCM. Pyridine (0.5 ml, 6.2 mmol) and DMAP (76 mg, 0.6 mmol) were then added. Afterwards, AB_2C anhydride monomer (3.0 g, 4.2 mmol) was added to the reaction mixture under vigorous stirring. The reaction was allowed to proceed overnight and monitored by NMR as well as MALDI-TOF-MS. Upon completion the crude reaction was precipitated three times in ether and the product was isolated by filtration to obtain **PEG20k-G3-(ally)$_8$(ac)$_8$** as a white powder (5.1 g, 84 %). ^{1}H NMR ($CDCl_3$, 400 MHz), δ/ppm: 5.86-5.70 (8H, m, H23), 5.11-4.92 (16H, m, H24), 4.41-4.01 (88H, m, H4, H8, H12, H16, H19), 3.64 (1818H, s, PEG), 2.48-2.27 (32H, m, H21, H22), 1.40 (24H, s, H18), 1.34 (24H, s, H18), 1.24 (42H, m, H3, H7, H11), 1.13 (24H, s, H15). ^{13}C NMR ($CDCl_3$, 101 MHz), δ/ppm: 173.6-171.7 (C1, C5, C9, C13, C20), 136.5 (C23), 115.7 (C24), 98.1 (C17), 70.6 (PEG), 68.7 (PEG), 68.3 (PEG), 66.0-63.6 (C4, C8, C12, C16, C19), 46.7-46.6 (C6, C10, C14), 42.1 (C2), 33.2 (C21), 28.7 (C22), 25.2 (C18), 22.1 (C18), 18.5-17.5 (C3, C7, C11, C15). SEC (DMF) M_n =28547 g mol^{-1}, M_w= 30735 g mol^{-1}, Đ = 1.07.

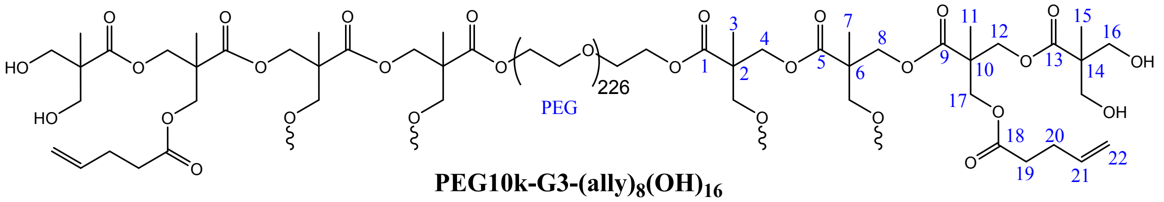

PEG10k-G3-(ally)$_8$(OH)$_{16}$

PEG10k-G3-(ally)$_8$(OH)$_{16}$. Dowex™ (3 g) was added to a solution of **PEG10k-G3-(ally)$_8$(ac)$_8$** (2.97 g, 0.22 mmol) in DCM/MeOH mixture (10 mL). The reaction was allowed to proceed with stirring at room temperature. Progress was monitored with ^{1}H NMR spectroscopy. After completion, the Dowex™ was removed by filtration and the filtrate was evaporated. The resulting product was dissolved in DCM, precipitated in ether and dried *in vacuo* to give **PEG10k-G3-(ally)$_8$(OH)$_{16}$** as a white powder (2.50 g, 86%). ^{1}H NMR ($CDCl_3$, 400 MHz), δ/ppm: 5.82-5.70 (8H, m, H21), 5.08-4.92 (16H, m, H22), 4.32-4.13 (56H, m, H4, H8, H12, H17), 3.90-3.41 (942H, s, PEG, H16), 2.47-2.25 (32H, m, H19, H20), 1.28-1.17 (42H, m, H3, H7, H11), 1.04 (24H, s, H15). ^{13}C NMR ($CDCl_3$, 101 MHz), δ/ppm: 174.8-172.1 (C1, C5, C9, C13, C18), 136.3 (C21), 115.6 (C22), 70.4 (PEG), 68.6 (PEG), 68.2 (PEG), 66.7-63.5 (C4, C8, C12, C17), 49.7 (C16), 46.5-46.4 (C6, C10, C14), 33.1 (C19), 28.5 (C20), 17.6-17.1 (C3, C7, C11, C15). SEC (DMF) M_n =14934 g mol^{-1}, M_w= 15305 g mol^{-1}, Đ = 1.02.

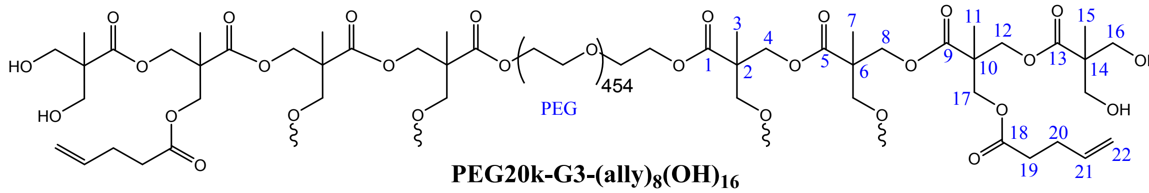

PEG20k-G3-(ally)$_8$(OH)$_{16}$

PEG20k-G3-(ally)$_8$(OH)$_{16}$. Dowex™ (3 g) was added to a solution of **PEG20k-G3-(ally)$_8$(ac)$_8$** (2.34 g, 0.1 mmol) in DCM/MeOH mixture (10 mL). The reaction was allowed to proceed with stirring at room temperature. Progress was monitored with ^{1}H NMR spectroscopy. After completion, the Dowex™ was removed by filtration and the filtrate was evaporated. The resulting product was dissolved in DCM, precipitated in ether and dried *in vacuo* to give **PEG20k-G3-(ally)$_8$(OH)$_{16}$** as a white powder (1.98 g, 86%). ^{1}H NMR ($CDCl_3$, 400 MHz), δ/ppm: 5.84-5.70 (8H, m, H21), 5.07-4.94 (16H, m, H22), 4.33-4.13 (56H, m, H4, H8, H12, H17), 3.83-3.46 (942H, s, PEG, H16), 2.46-2.28 (32H, m, H19, H20), 1.27-1.24 (42H, m, H3, H7, H11), 1.05 (24H, s, H15). ^{13}C NMR ($CDCl_3$, 101 MHz), δ/ppm: 174.9-172.6 (C1, C5, C9, C13, C18), 136.4 (C21), 115.7 (C22), 70.6 (PEG), 68.3 (PEG), 66.9-63.7 (C4, C8, C12, C17), 49.8 (C16), 46.7-46.4 (C6, C10, C14), 33.3 (C19), 28.7 (C20), 17.7-17.2 (C3, C7, C11, C15). SEC (DMF) M_n =24764 g mol^{-1}, M_w= 25820 g mol^{-1}, Đ = 1.04.

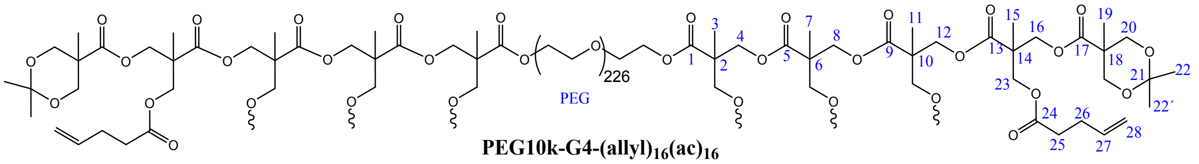

PEG10k-G4-(allyl)$_{16}$(ac)$_{16}$

PEG10k-G4-(allyl)$_{16}$(ac)$_{16}$. In a RBF equipped with a magnetic stirrer **PEG10k-G3-(OH)$_{16}$** (2.5 g, 0.2 mmol) was dissolved in DCM. Pyridine (0.6 ml, 6.9 mmol) and DMAP (98 mg, 0.7 mmol) were then added. Afterwards, AB_2C anhydride monomer (5.8 g, 6.9 mmol) was added to the reaction mixture under vigorous stirring. The reaction was allowed to proceed overnight and monitored by NMR as well as MALDI-TOF-MS. Upon completion the crude reaction was precipitated three times in ether and the product was isolated by filtration to obtain **PEG10k-G4-(allyl)$_{16}$(ac)$_{16}$** as a white powder (3.6 g, 98 %). ^{1}H NMR ($CDCl_3$, 400 MHz), δ/ppm: 5.85-5.69 (16H, m, H27), 5.08-4.92 (32H, m, H28), 4.36-4.06 (184H, m, H4, H8, H12, H16, H20, H23), 3.62 (910H, s, PEG), 2.45-2.25 (64H, m, H25, H26), 1.39 (48H, s, H22), 1.32 (48H, s, H22), 1.22 (90H, m, H3, H7, H11, H15), 1.11 (48H, s, H19). ^{13}C NMR ($CDCl_3$, 101 MHz), δ/ppm: 173.7-171.4 (C1, C5, C9, C13, C17, C24), 136.6 (C27), 115.7 (C28), 98.2 (C21), 70.6 (PEG), 68.7 (PEG), 68.3 (PEG), 66.1-63.6 (C4, C8, C12, C16, C20, C23), 46.8-46.6 (C6, C10,

C14, C18), 42.1 (C2), 33.3 (C25), 28.7 (C26), 25.3 (C22), 22.1 (C22), 18.7-17.3 (C3, C7, C11, C15, C19). SEC (DMF) M_n =14519 g mol^{-1}, M_w= 14890 g mol^{-1}, Đ = 1.03.

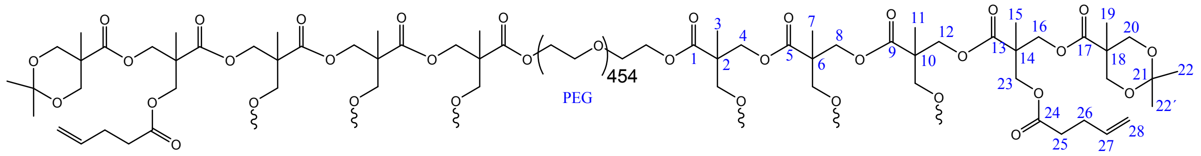

PEG20k-G4-(allyl)$_{16}$(ac)$_{16}$

PEG20k-G4-(allyl)$_{16}$(ac)$_{16}$. In a RBF equipped with a magnetic stirrer **PEG20k-G3-(OH)$_{16}$** (3.0 g, 0.14 mmol) was dissolved in DCM. Pyridine (0.4 ml, 4.4 mmol) and DMAP (54 mg, 0.4 mmol) were then added. Afterwards, AB_2C anhydride monomer (3.2 g, 4.4 mmol) was added to the reaction mixture under vigorous stirring. The reaction was allowed to proceed overnight and monitored by NMR as well as MALDI-TOF-MS. Upon completion the crude reaction was precipitated three times in ether and the product was isolated by filtration to obtain **PEG20k-G4-(allyl)$_{16}$(ac)$_{16}$** as a white and sticky powder (3.7 g, 99 %). ^{1}H NMR ($CDCl_3$, 400 MHz), δ/ppm: 5.85-5.69 (16H, m, H27), 5.08-4.92 (32H, m, H28), 4.35-4.05 (184H, m, H4, H8, H12, H16, H20, H23), 3.62 (1818H, s, PEG), 2.45-2.25 (64H, m, H25, H26), 1.39 (48H, s, H22), 1.33 (48H, s, H22′), 1.23 (90H, m, H3, H7, H11, H15), 1.12 (48H, s, H19). ^{13}C NMR ($CDCl_3$, 101 MHz), δ/ppm: 174.2-171.2 (C1, C5, C9, C13, C17, C24), 136.5 (C27), 115.7 (C28), 98.1 (C21), 70.6 (PEG), 68.7 (PEG), 68.3 (PEG), 66.7-63.3 (C4, C8, C12, C16, C20, C23), 46.9-46.3 (C6, C10, C14, C18), 42.0 (C2), 33.2 (C25), 28.7 (C26), 25.3 (C22), 22.0 (C22), 18.7-17.3 (C3, C7, C11, C15, C19). SEC (DMF) M_n =27186 g mol^{-1}, M_w= 27941 g mol^{-1}, Đ = 1.03.

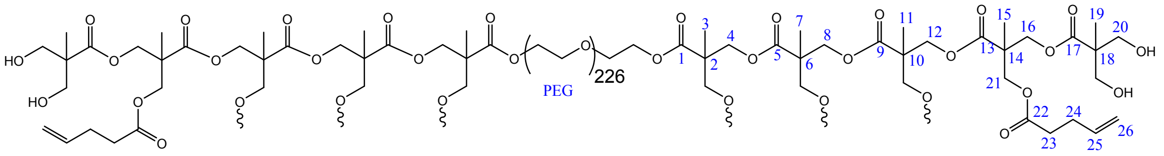

PEG10k-G4-(allyl)$_{16}$(OH)$_{32}$

PEG10k-G4-(allyl)$_{16}$(OH)$_{32}$. Dowex™ (2 g) was added to a solution of **PEG10k-G4-(allyl)$_{16}$(ac)$_{16}$** (2.5 g, 0.14 mmol) in DCM/MeOH mixture (10 mL). The reaction was allowed to proceed with stirring at room temperature. Progress was monitored with ^{1}H NMR spectroscopy. After completion, the Dowex™ was removed by filtration and the filtrate was evaporated. The resulting product was dissolved in DCM, precipitated in ether and dried *in vacuo* to give **PEG10k-G4-(allyl)$_{16}$(OH)$_{32}$** as a white powder (2.1 g, 88%).^{1}H NMR ($CDCl_3$, 400 MHz), δ/ppm: 5.84-5.69 (16H, m, H25), 5.08-4.87 (32H, m, H26), 4.39-4.08 (120H, m, H4, H8, H12, H16, H21), 3.81-3.41 (974H, s, PEG, H20), 2.45-2.23 (64H, m, H23, H24), 1.30-

1.12 (90H, m, H3, H7, H11, H15), 1.04 (48H, s, H19). ^{13}C NMR ($CDCl_3$, 101 MHz), δ/ppm: 175.3-171.7 (C1, C5, C9, C13, C17, C22), 136.4 (C25), 115.8 (C26), 70.6 (PEG), 68.8 (PEG), 66.8-63.3 (C4, C8, C12, C16, C21), 49.8 (C20), 46.9-46.1 (C6, C10, C14, C18), 33.3 (C23), 28.7 (C24), 18.0-16.6 (C3, C7, C11, C15, C19). SEC (DMF) M_n =18171 g mol^{-1}, M_w= 14612 g mol^{-1}, Đ = 1.02.

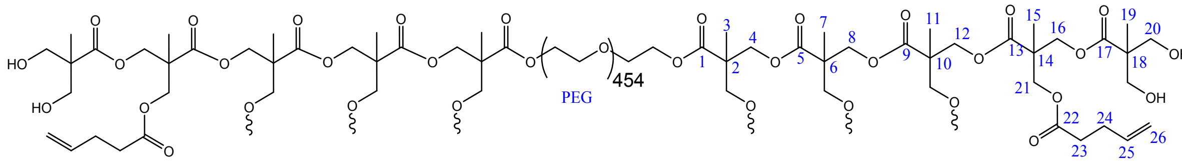

PEG20k-G4-(allyl)$_{16}$(OH)$_{32}$

PEG20k-G4-(allyl)$_{16}$(OH)$_{32}$. Dowex™ (3 g) was added to a solution of **PEG20k-G4-(allyl)$_{16}$(ac)$_{16}$** (3.0 g, 0.1 mmol) in DCM/MeOH mixture (10 mL). The reaction was allowed to proceed with stirring at room temperature. Progress was monitored with ^1H NMR spectroscopy. After completion, the Dowex™ was removed by filtration and the filtrate was evaporated. The resulting product was dissolved in DCM, precipitated in ether and dried *in vacuo* to give **PEG20k-G4-(allyl)$_{16}$(OH)$_{32}$** as a white powder (2.5 g, 86%). ^1H NMR ($CDCl_3$, 400 MHz), δ/ppm: 5.84-5.69 (16H, m, H25), 5.09-4.93 (32H, m, H26), 4.34-4.11 (120H, m, H4, H8, H12, H16, H21), 3.84-3.38 (974H, s, PEG, H20), 2.46-2.23 (64H, m, H23, H24), 1.31-1.17 (90H, m, H3, H7, H11, H15), 1.06 (48H, s, H19).^{13}C NMR ($CDCl_3$, 101 MHz), δ/ppm: 174.8-170.9 (C1, C5, C9, C13, C17, C22), 136.5 (C25), 115.8 (C26), 70.6 (PEG), 68.8 (PEG), 66.8-64.0 (C4, C8, C12, C16, C21), 49.8 (C20), 46.9-46.2 (C6, C10, C14, C18), 33.3 (C23), 28.7 (C24), 18.0-16.9 (C3, C7, C11, C15, C19). SEC (DMF) M_n =29448 g mol^{-1}, M_w= 30244 g mol^{-1}, Đ = 1.03.

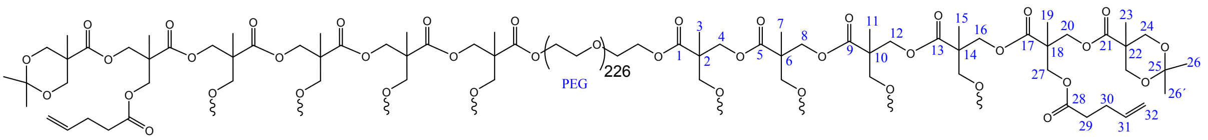

PEG10k-G5-(allyl)$_{32}$(ac)$_{32}$

PEG10k-G5-(allyl)$_{32}$(ac)$_{32}$. In a RBF equipped with a magnetic stirrer **PEG10k-G4-(OH)$_{32}$** (1.0 g, 0.07 mmol) was dissolved in DCM. Pyridine (0.4 ml, 4.5 mmol) and DMAP (55 mg, 0.45 mmol) were then added. Afterwards, AB_2C anhydride monomer (3.3 g, 4.5 mmol) was added to the reaction mixture under vigorous stirring. The reaction was allowed to proceed overnight and monitored by NMR as well as MALDI-TOF-MS. Upon completion the crude reaction was precipitated three times in ether and the product was isolated by filtration to obtain **PEG10k-G5-(allyl)$_{32}$(ac)$_{32}$** as a white and sticky powder (0.986 g, 60 %). ^1H NMR ($CDCl_3$,

400 MHz), δ/ppm: 5.84-5.71 (32H, m, H31), 5.10-4.91 (64H, m, H32), 4.37-4.07 (376H, m, H4, H8, H12, H16, H20, H24, H27), 3.64 (910H, s, PEG), 2.47-2.27 (128H, m, H31, H32), 1.40 (96H, s, H26), 1.33 (96H, s, H26′), 1.28-1.19 (186H, s, H3, H7, H11, H15, H19), 1.13 (96H, s, H23).^{13}C NMR ($CDCl_3$, 101 MHz), δ/ppm:. 173.9-171.5 (C1, C5, C9, C13, C17, C21, C28), 136.5 (C31), 115.9 (C32), 96.9 (C25), 70.7 (PEG), 66.8-64.3 (C4, C8, C12, C16, C20, C24, C27), 46.8-46.6 (C6, C10, C14, C18, C22), 33.3 (C29), 28.8 (C30), 26.2 (C26), 18.0-17.3 (C3, C7, C11, C15, C19, C23). SEC (DMF) M_n =17802 g mol^{-1}, M_w= 18172 g mol^{-1}, Đ = 1.02.

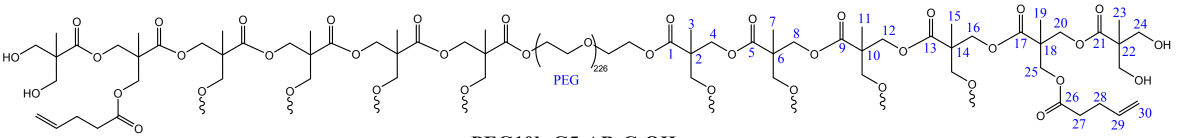

PEG10k-G5-AB$_2$C-OH

PEG10k-G5-(allyl)$_{32}$(OH)$_{64}$. Dowex™ (1.0 g) was added to a solution of **PEG10k-G5-(allyl)$_{32}$(ac)$_{32}$** (0.9 g, 0.04 mmol) in DCM/MeOH mixture. The reaction was allowed to proceed with stirring at room temperature. Progress was monitored with ^{1}H NMR spectroscopy. After completion, the Dowex™ was removed by filtration and the filtrate was evaporated. The resulting product was dissolved in DCM, precipitated in ether and dried *in vacuo* to give **PEG10k-G5-(allyl)$_{32}$(OH)$_{64}$** as a white powder (0.649 g, 76 %). ^{1}H NMR ($CDCl_3$, 400 MHz), δ/ppm: 5.84-5.71 (32H, m, H29), 5.09-4.93 (64H, m, H30), 4.38-4.12 (248H, m, H4, H8, H12, H16, H20, H25), 3.63 (1038H, s, PEG, H24), 2.47-2.27 (128H, m, H27, H28), 1.32-1.16 (186H, s, H3, H7, H11, H15, H19), 1.06 (96H, s, H23).^{13}C NMR ($CDCl_3$, 101 MHz), δ/ppm: 176.1-171.6 (C1, C5, C9, C13, C17, C21, C26), 136.6 (C29), 115.8 (C30), 70.7 (PEG), 67.3-63.9 (C4, C8, C12, C16, C20, C25), 49.9 (C24), 48.7-46.7 (C6, C10, C14, C18, C22), 33.4 (C27), 28.7 (C28), 18.2-16.9 (C3, C7, C11, C15, C19, C23). SEC (DMF) M_n =22590 g mol^{-1}, M_w= 23073 g mol^{-1}, Đ = 1.02.

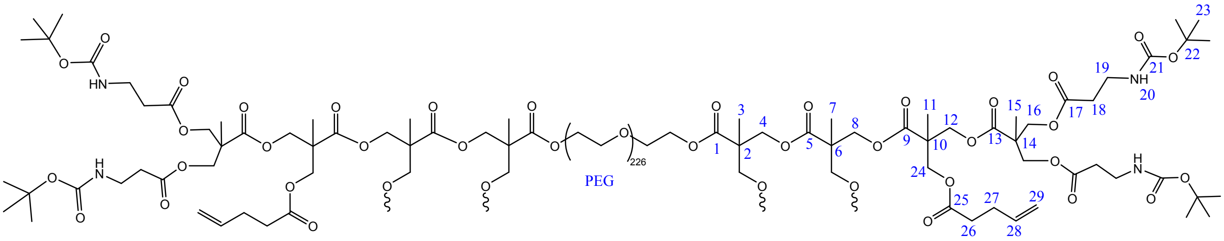

PEG10k-G3-(allyl)$_8$(NHBoc)$_{16}$

PEG10k-G3-(allyl)$_8$(NHBoc)$_{16}$. In a RBF equipped with a magnetic stirrer **PEG10k-G3-(allyl)$_8$(OH)$_{16}$** (0.988 g, 0.075 mmol) was dissolved in DCM. Pyridine (0.3 ml, 3.6 mmol) and DMAP (0.0462 g, 0.378 mmol) were then added. Afterwards, Boc β-alanine anhydride (0.865

g, 2.4 mmol) was added to the reaction mixture under vigorous stirring. The reaction was allowed to proceed overnight and monitored by NMR. After completion, the product was isolated by precipitation three times in ether to obtain **PEG10k-G3-(allyl)$_8$(NHBoc)$_{16}$** as a white powder (0.820 g, 66 %). ^{1}H NMR ($CDCl_3$, 400 MHz), δ/ppm: 5.85-5.71 (8H, m, H28), 5.20 (16H, s, H20), 5.08-4.94 (16H, m, H29), 4.34-4.08 (88 H, m, H4, H8, H12, H16, H24), 3.63 (910H, s, PEG), 3.36 (32H, m, H18), 2.52 (32H, m, H19), 2.45-2.28 (32H, m, H26, H27), 1.42 (144H, s, H23), 1.30-1.17 (66H, s, H3, H7, H11, H15). ^{13}C NMR ($CDCl_3$, 101 MHz), δ/ppm: 172.4-171.6 (C1, C5, C9, C13, C17, C25), 155.8 (C21), 136.4 (C28), 115.7 (C29), 79.3 (C22), 70.6 (PEG),68.7 (PEG), 65.5-64.6 (C4, C8, C12, C16, C24), 46.7-46.3 (C6, C10, C14), 36.1 (C19), 34.4 (C18), 33.1 (C26), 28.6 (C27), 28.4 (C23), 18.0-17.3 (C3, C7, C11, C15). SEC (DMF) M_n =15900 g mol^{-1}, M_w= 16263 g mol^{-1}, Đ = 1.02.

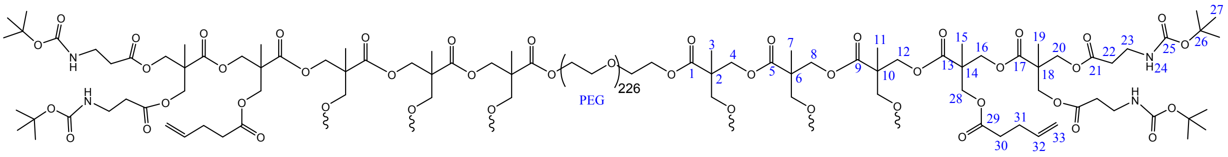

PEG10k-G4-(allyl)$_{16}$(NHBoc)$_{32}$

PEG10k-G4-(allyl)$_{16}$(NHBoc)$_{32}$. In a RBF equipped with a magnetic stirrer **PEG10k-G4-(allyl)$_{16}$(OH)$_{32}$** (1.0 g, 0.060 mmol) was dissolved in DCM. Pyridine (0.3 ml, 3.6 mmol) and DMAP (0.047 g, 0.384 mmol) were then added. Afterwards, Boc β-alanine anhydride (1.4 g, 4.0 mmol) was added to the reaction mixture under vigorous stirring. The reaction was allowed to proceed overnight and monitored by NMR. After completion, the product was isolated by precipitation three times in ether to obtain **PEG10k-G4-(allyl)$_{16}$(NHBoc)$_{32}$** as a white powder (0.937 g, 71 %). ^{1}H-NMR ($CDCl_3$, 400 MHz), δ/ppm: 5.84-5.68 (16H, m, H32), 5.24 (32H, s, H24), 5.08-4.91 (32H, m, H33), 4.34-4.07 (184 H, m, H4, H8, H12, H16, H20, H28), 3.62 (910H, s, PEG), 3.34 (64H, m, H22), 2.51 (64H, m, H23), 2.44-2.26 (64H, m, H30, H31), 1.41 (288H, s, H27), 1.28-1.19 (138H, s, H3, H7, H11, H15, H19). ^{13}C-NMR ($CDCl_3$, 101 MHz), δ/ppm: 172.2-171.4 (C1, C5, C9, C13, C17, C21, C29), 155.8 (C25), 136.4 (C32), 115.7 (C33), 79.3 (C26), 70.5 (PEG),68.5 (PEG), 65.3-64.7 (C4, C8, C12, C16, C20, C28), 46.6-46.4 (C6, C10, C14, C18), 36.0 (C23), 34.4 (C22), 33.1 (C30), 28.6 (C31), 28.3 (C27), 18.0-17.1 (C3, C7, C11, C15, C19). SEC (DMF) M_n =17858 g mol^{-1}, M_w= 18209 g mol^{-1}, Đ = 1.02.

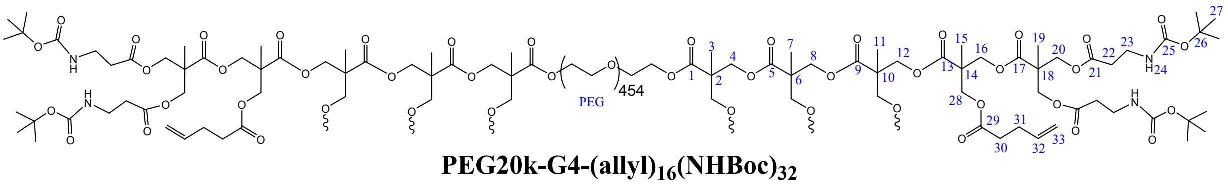

PEG20k-G4-(allyl)$_{16}$(NHBoc)$_{32}$

PEG20k-G4-(allyl)$_{16}$(NHBoc)$_{32}$. In a RBF equipped with a magnetic stirrer **PEG20k-G4-(allyl)$_{16}$(OH)$_{32}$** (1.0 g, 0.038 mmol) was dissolved in DCM. Pyridine (0.2 ml, 2.6 mmol) and DMAP (0.029 g, 0.241 mmol) were then added. Afterwards, Boc β-alanine anhydride (0.865 g, 2.4 mmol) was added to the reaction mixture under vigorous stirring. The reaction was allowed to proceed overnight and monitored by NMR. After completion, the product was isolated by precipitation three times in ether to obtain **PEG20k-G4-(allyl)$_{16}$(NHBoc)$_{32}$** as a white powder (0.874 g, 72 %). ^{1}H-NMR ($CDCl_3$, 400 MHz), δ/ppm: 5.85-5.68 (16H, m, H32), 5.23 (32H, s, H24), 5.09-4.91 (32H, m, H33), 4.36-4.06 (184 H, m, H4, H8, H12, H16, H20, H28), 3.63 (1818H, s, PEG), 3.36 (64H, m, H22), 2.52 (64H, m, H23), 2.47-2.26 (64H, m, H30, H31), 1.41 (288H, s, H27), 1.34-1.13 (138H, s, H3, H7, H11, H15, H19). ^{13}C-NMR ($CDCl_3$, 101 MHz), δ/ppm: 173.3-171.2 (C1, C5, C9, C13, C17, C21, C29), 156.0 (C25), 136.5 (C32), 115.8 (C33), 79.6 (C26), 70.7 (PEG),68.7 (PEG), 65.7-64.5 (C4, C8, C12, C16, C20, C28), 46.9-46.3 (C6, C10, C14, C18), 36.2 (C23), 34.5 (C22), 33.2 (C30), 28.7 (C31), 28.5 (C27), 18.1-17.3 (C3, C7, C11, C15, C19). SEC (DMF) M_n =29012 g mol^{-1}, M_w= 29910 g mol^{-1}, Đ = 1.03.

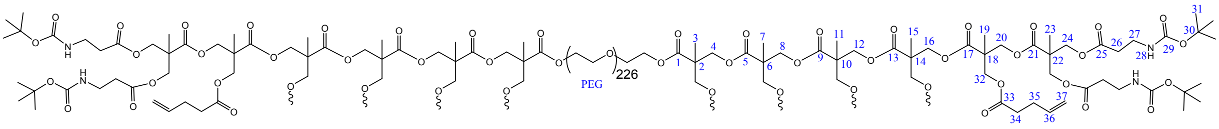

PEG10k-G5-(allyl)$_{32}$(NHBoc)$_{64}$

PEG10k-G5-(allyl)$_{32}$(NHBoc)$_{64}$. In a RBF equipped with a magnetic stirrer **PEG10k-G5-(allyl)$_{32}$(OH)$_{64}$** (1.35 g, 0.057 mmol) was dissolved in DCM. Pyridine (0.6 ml, 7.3 mmol) and DMAP (0.089 g, 0.73 mmol) were then added. Afterwards, Boc β-alanine anhydride (2.6 g, 7.3 mmol) was added to the reaction mixture under vigorous stirring. The reaction was allowed to proceed overnight and monitored by NMR. After completion, the product was isolated by precipitation three times in ether to obtain **PEG10k-G5-(allyl)$_{32}$(NHBoc)$_{64}$** as a white powder (1.45 g, 74%). ^{1}H-NMR ($CDCl_3$, 400 MHz), δ/ppm: 5.84-5.68 (32H, m, H36), 5.26 (64H, s, H27), 5.08-4.91 (64H, m, H37), 4.37-4.05 (376 H, m, H4, H8, H12, H16, H20, H24, H32), 3.62 (910H, s, PEG), 3.33 (128H, m, H26), 2.51 (128H, m, H27), 2.44-2.25 (128H, m, H34, H35), 1.40 (576H, s, H31), 1.30-1.15 (282H, s, H3, H7, H11, H15, H19, H23). ^{13}C-NMR ($CDCl_3$, 101 MHz), δ/ppm: 172.8-171.2 (C1, C5, C9, C13, C17, C21, C25, C33), 155.9 (C29), 136.5 (C36), 115.8 (C37), 79.4 (C30), 70.7 (PEG), 65.9-64.0 (C4, C8, C12, C16, C20, C24, C32), 46.9-46.3 (C6, C10, C14, C18, C22), 36.2 (C27), 34.5 (C26), 33.2 (C34), 28.7 (C35), 28.5 (C31), 18.1-17.5 (C3, C7, C11, C15, C19, C23). SEC (DMF) M_n =31137 g mol^{-1}, M_w= 42509 g mol^{-1}, Đ = 1.36.

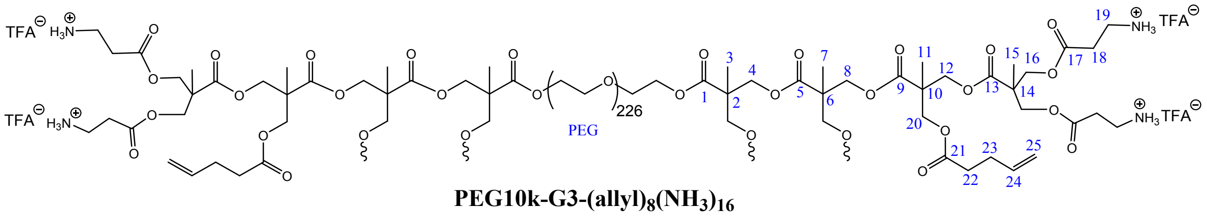

PEG10k-G3-(allyl)$_8$(NH$_3$)$_{16}$

PEG10k-G3-(allyl)$_8$(NH$_3$$^+$)$_{16}$. **PEG10k-G3-(allyl)$_8$(NHBoc)$_{16}$** (0.2 g, 0.125 mmol) was dissolved in DCM with stirring at room temperature. Trifluoroacetic acid (TFA) was then added carefully (0.5 mL). The vessel was then sealed with a septum equipped with a needle to release the CO_2 generated by the reaction. The reaction mixture was stirred for 1 h and progress was monitored with ^{1}H NMR spectroscopy. Upon completion the solvents were removed and the product was dried *in vacuo* to give **PEG10k-G3-(allyl)$_8$(NH$_3$$^+$)$_{16}$** (158 mg, 79 %). ^{1}H NMR (CD$_3$OD, 400 MHz), δ/ppm: 5.88-5.75 (8H, m, H24), 5.08-4,92 (16H, m, H25), 4.43-4.12 (88 H, m, H4, H8, H12, H16, H20), 3.61 (910H, s, PEG), 3.22 (32H, m, H18), 2.79 (32H, m, H19), 2.49-2.27 (32H, m, H22, H23), 1.36-1.16 (66H, m, H3, H7, H11, H15). ^{13}C NMR (CD$_3$OD, 101 MHz), δ/ppm: 173.9-171.8 (C1, C5, C9, C13, C17, C21, TFA), 163.0-162.6 (TFA); 138.0 (C24), 116.3 (C25), 71.5 (PEG), 66.9-66.2 (C4, C8, C12, C16, C20), 49.9-47.7 (C6, C10, C14), 36.6 (C19), 34.2 (C18), 32.2 (C22), 29.9 (C23), 18.5-18.1 (C3, C7, C11, C15).

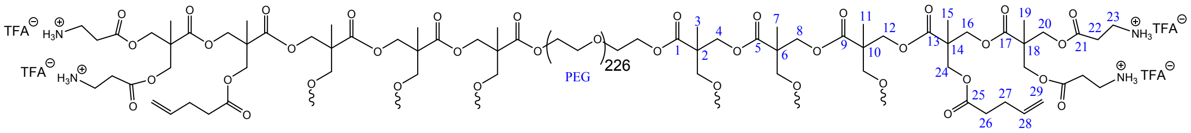

PEG10k-G4-(allyl)$_{16}$(NH$_3$)$_{32}$

PEG10k-G4-(allyl)$_{16}$(NH$_3$$^+$)$_{32}$. **PEG10k-G4-(allyl)$_{16}$(NHBoc)$_{32}$** (0.5 g, 0.023 mmol) was dissolved in DCM with stirring at room temperature. Trifluoroacetic acid (TFA) was then added carefully (0.6 mL). The vessel was then sealed with a septum equipped with a needle to release the CO_2 generated by the reaction. The reaction mixture was stirred for 1 h and progress was monitored with ^{1}H NMR spectroscopy. Upon completion the solvents were removed and the product was dried *in vacuo* to give **PEG10k-G4-(allyl)$_{16}$(NH$_3$$^+$)$_{32}$** (0.320 g, 63 %). ^{1}H NMR (CD$_3$OD, 400 MHz), δ/ppm: 5.90-5.75 (16H, m, H28), 5.12-4.95 (32H, m, H29), 4.45-4.14 (184 H, m, H4, H8, H12, H16, H20, H24), 3.65 (910H, s, PEG), 3.29 (64H, m, H22), 2.82 (64H, m, H23), 2.51-2.28 (64H, m, H26, H27), 1.41-1.20 (138H, m, H3, H7, H11, H15, H19). ^{13}C NMR (CD$_3$OD, 101 MHz), δ/ppm: 174.4-171.2 (C1, C5, C9, C13, C17, C21, C25, TFA), 163.3-161.7 (TFA), 138.1 (C28), 116.5 (C29), 71.4 (PEG), 67.0-66.1 (C4, C8, C12, C16, C20, C24), 49.9-47.7 (C6, C10, C14, C18), 36.5 (C23), 34.2 (C22), 32.2 (C26), 29.9 (C27), 18.7-18.1 (C3, C7, C11, C15, C19).

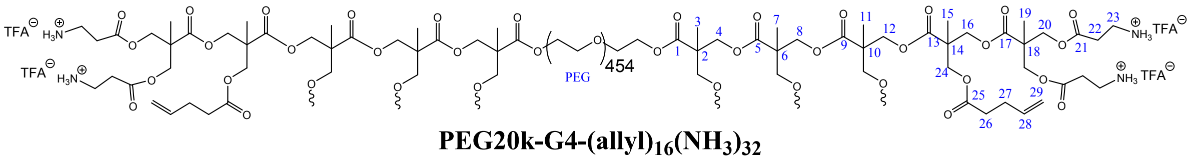

PEG20k-G4-(allyl)$_{16}$(NH$_3$)$_{32}$

PEG20k-G4-(allyl)$_{16}$(NH$_3^+$)$_{32}$. **PEG20k-G4-(allyl)$_{16}$(NHBoc)$_{32}$** (0.5 g, 0.016 mmol) was dissolved in DCM with stirring at room temperature. Trifluoroacetic acid (TFA) was then added carefully (0.6 mL). The vessel was then sealed with a septum equipped with a needle to release the CO_2 generated by the reaction. The reaction mixture was stirred for 1 h and progress was monitored with ^{1}H NMR spectroscopy. Upon completion the solvents were removed and the product was dried *in vacuo* to give **PEG20k-G4-(allyl)$_{16}$(NH$_3^+$)$_{32}$** (0.412 mg, 79 %). ^{1}H NMR (CD$_3$OD, 400 MHz), δ/ppm: 5.89-5.76 (16H, m, H28), 5.11-4.95 (32H, m, H29), 4.42-4.15 (184 H, m, H4, H8, H12, H16, H20, H24), 3.64 (1818H, s, PEG), 3.29 (64H, m, H23), 2.82 (64H, m, H22), 2.50-2.29 (64H, m, H26, H27), 1.38-1.23 (138H, m, H3, H7, H11, H15, H19). ^{13}C NMR (CD$_3$OD, 101 MHz), δ/ppm: 174.1-171.2 (C1, C5, C9, C13, C17, C21, C25, TFA), 163.3-161.7 (TFA), 138.1 (C28), 116.4 (C29), 71.4 (PEG), 67.2-65.8 (C4, C8, C12, C16, C20, C24), 49.9-47.7 (C6, C10, C14, C18), 36.6 (C23), 34.2 (C22), 32.2 (C26), 29.9 (C27), 18.8-17.8 (C3, C7, C11, C15, C19).

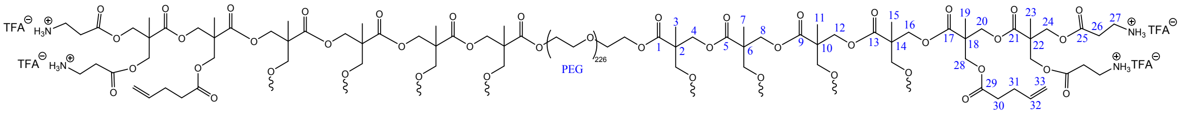

PEG10k-G5-AB$_2$C-B-alanine-NH$_3^+$

PEG10k-G5-(allyl)$_{32}$(NH$_3^+$)$_{64}$. **PEG10k-G5-(allyl)$_{32}$(NHBoc)$_{64}$** (0.5 g, 0.014 mmol) was dissolved in DCM with stirring at room temperature. Trifluoroacetic acid (TFA) was then added carefully (0.7 mL). The vessel was then sealed with a septum equipped with a needle to release the CO_2 generated by the reaction. The reaction mixture was stirred for 1 h and progress was monitored with ^{1}H NMR spectroscopy. Upon completion the solvents were removed and the product was dried *in vacuo* to give **PEG10k-G5-(allyl)$_{32}$(NH$_3^+$)$_{64}$** (0.466 mg, 94 %). ^{1}H NMR (CD$_3$OD, 400 MHz), δ/ppm: 5.94-5.76 (32H, m, H32), 5.13-5.00 (64H, m, H33), 4.47-4.13 (376H, m, H4, H8, H12, H16, H20, H24, H28), 3.67 (910H, s, PEG), 3.26 (128H, m, H27), 2.83 (128H, m, H26), 2.49-2.29 (128H, m, H30, H31), 1.38-1.23 (282H, m, H3, H7, H11, H15, H19, H23). ^{13}C NMR (CD$_3$OD, 101 MHz), δ/ppm: 174.1-171.4 (C1, C5, C9, C13, C17, C21, C25, C29, TFA), 163.3-161.0 (TFA), 138.1 (C32), 116.2 (C33), 71.2 (PEG), 67.7-65.9 (C4, C8, C12, C16, C20, C24, C28), 49.9-47.7 (C6, C10, C14, C18, C22), 36.5 (C27), 34.2 (C26), 32.2 (C30), 29.9 (C31), 18.8-17.9 (C3, C7, C11, C15, C19, C23).

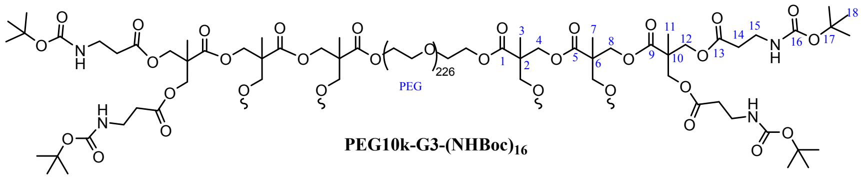

PEG10k-G3-(NHBoc)$_{16}$

PEG10k-G3-(NHBoc)$_{16}$. In a RBF equipped with a magnetic stirrer Boc β-alanine acid (0.94 g, 5.0 mmol) was activated with CDI (0.8 g, 5.0 mmol) during 1 h in DCM as solvent at room temperature. Afterwards, **PEG10k-G3-(OH)$_{16}$** (1.8 g, 0.16 mmol) and CsF (77.8 mg, 0.5 mmol) were added. The reaction was allowed to proceed overnight and monitored by NMR. After completion, the product was isolated by precipitation three times in ether to obtain **PEG10k-G3-(NHBoc)$_{16}$** as a white powder (2.0 g, 87 %). ^{1}H-NMR ($CDCl_3$, 400 MHz), δ/ppm: 5.26 (8H, s, NH), 4.30-4.11 (56H, m, H4, H8 and H12), 3.6 (910H, s, PEG) 3.35 (32H, m, H14), 2.51 (32H, m, H15), 1.41 (144H, s, H18), 1.30-1.20 (42H, m, H3, H7 and H11). ^{13}C-NMR ($CDCl_3$, 101 MHz), δ/ppm: 172.3-171.5 (C1, C5, C9, C13), 155.9 (C16), 79.4 (C17), 70.6 (PEG), 68.8 (PEG), 65.2-64.6 (C4, C8, C12), 46.7-46.5 (C2, C6, C10), 36.2 (C15), 34.5 (C14), 28.5 (C18), 18.2-17.2 (C3, C7, C11).

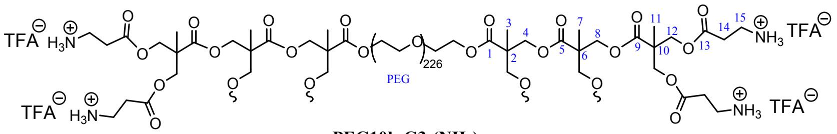

PEG10k-G3-(NH$_3$)$_{16}$

PEG10k-G3-(NH_3^+)$_{16}$. PEG10k-G3-(NHBoc)$_{16}$ (0.4 g, 0.028 mmol) was dissolved in DCM with stirring at room temperature. Trifluoroacetic acid (TFA) was then added carefully (0.6 mL). The vessel was then sealed with a septum equipped with a needle to release the CO_2 generated by the reaction. The reaction mixture was stirred for 1 h and progress was monitored with ^{1}H NMR spectroscopy. Upon completion the solvents were removed and the product was dried *in vacuo* to give **PEG10k-G3-(NH_3^+)$_{16}$.** (0.38 mg, 94 %). ^{1}H-NMR ($CDCl_3$, 400 MHz), δ/ppm: 4.32-4.24 (56H, m, H4, H8 and H12), 3.60 (910H, s PEG), 3.25 (32H, m, H15), 2.79 (32H, m, H14), 1.33-1.24 (42H, m, H3, H7 and H11). ^{13}C-NMR ($CDCl_3$, 101 MHz), δ/ppm: 172.0-171.7 (C1, C5, C9, C13), 160.8-169.6 (TFA), 66.7 (C4, C8, C12), 47.7 (C2, C6, C10), 36.6 (C15), 32.2 (C14), 18.24 (C2, C7, C11).

Figures

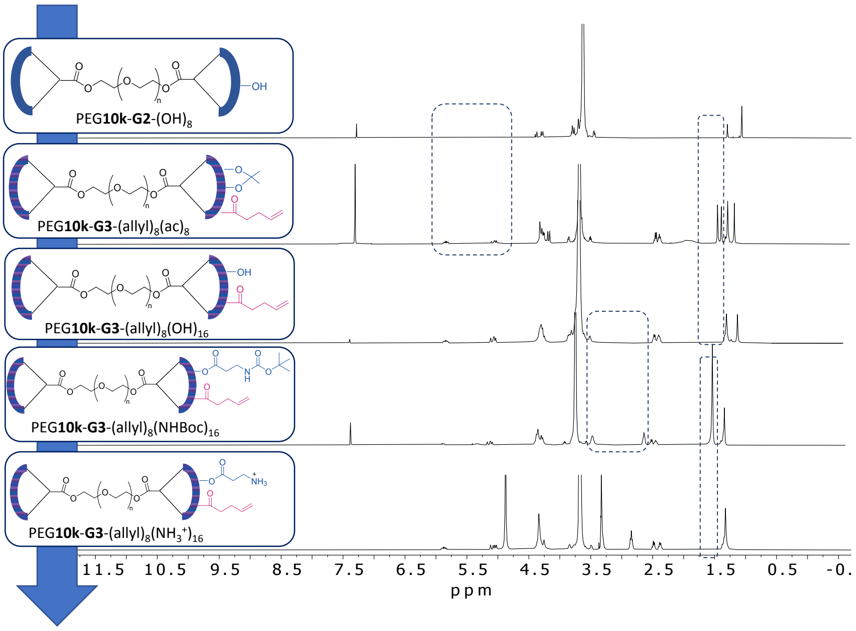

Figure S1. Reaction following-up by ^{1}H-NMR for the synthesis of the PEG10k-G3-(allyl)$_8$(NH$_3^+$)$_{16}$ starting from PEG10k-G2-(OH)$_8$.

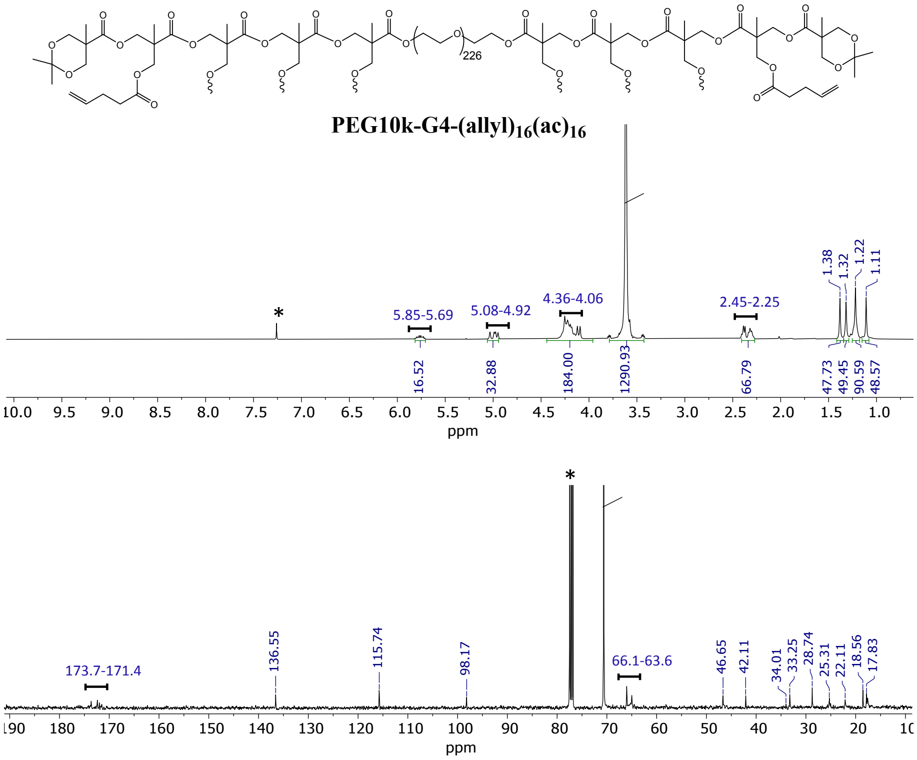

Figure S2. ^{1}H-NMR and ^{13}C-NMR in $CDCl_3$ (*) for PEG10k-G4-(allyl)$_{16}$(ac)$_{16}$.

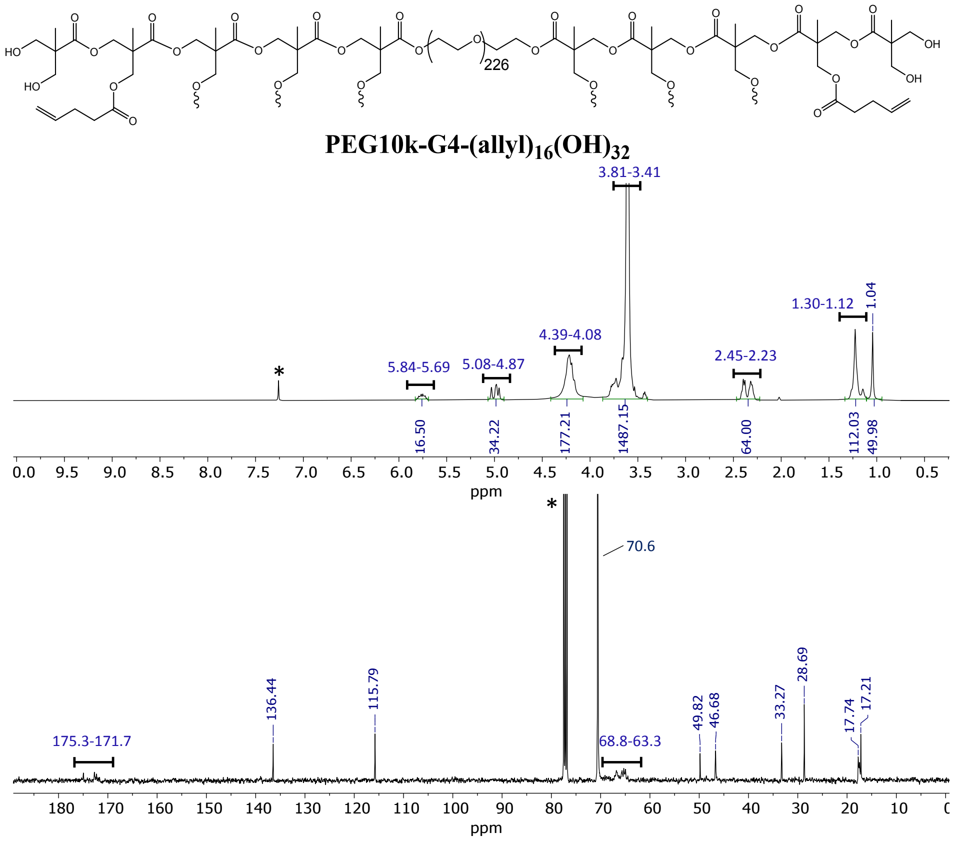

Figure S3. ^{1}H-NMR and ^{13}C-NMR in $CDCl_3$ (*) for PEG10k-G4-(allyl)$_{16}$(OH)$_{32}$.

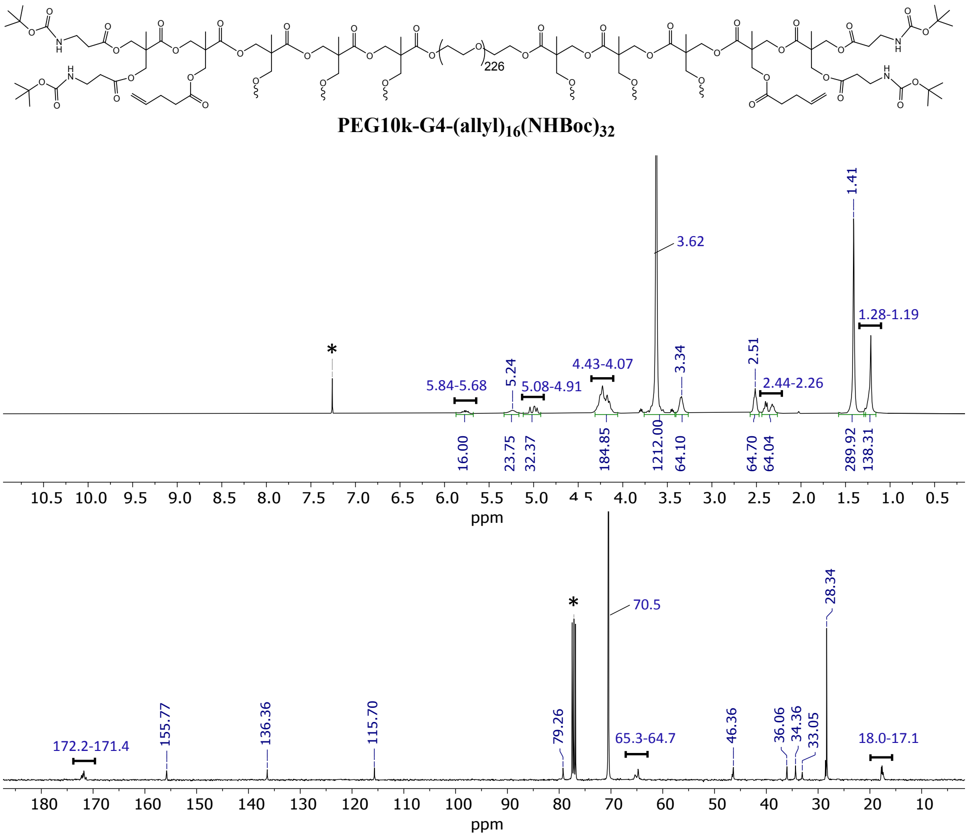

Figure S4. ^{1}H-NMR and ^{13}C-NMR in $CDCl_3$ (*) for PEG10k-G4-(allyl)$_{16}$(NHBoc)$_{32}$.

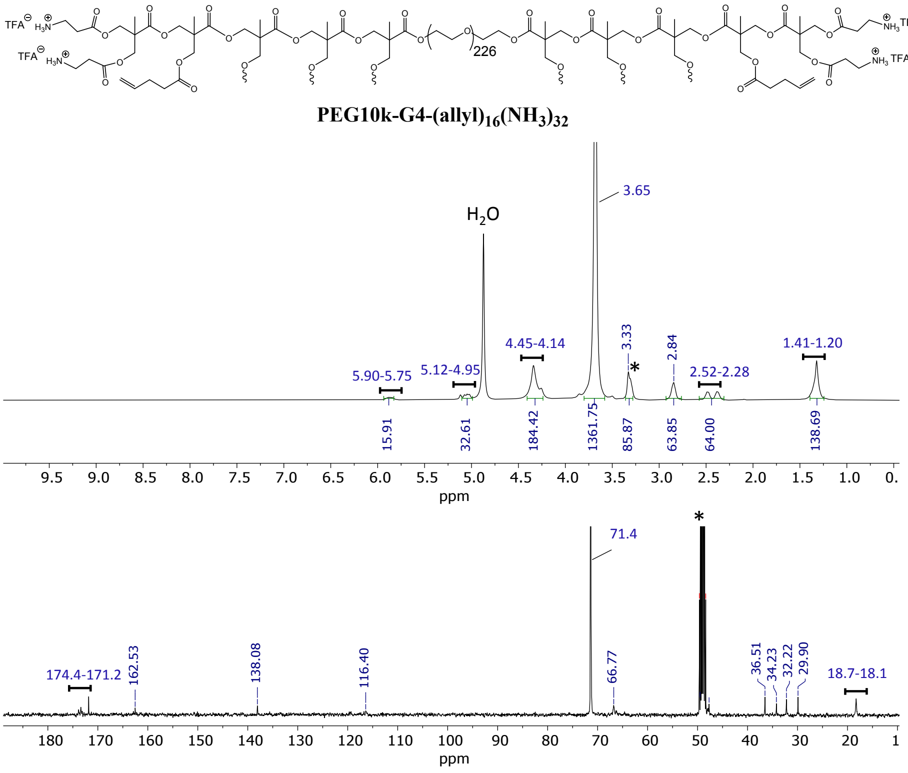

Figure S5. ^{1}H-NMR and ^{13}C-NMR in CD_3OD (*) for PEG10k-G4-(allyl)$_{16}$(NH_3^+)$_{32}$.

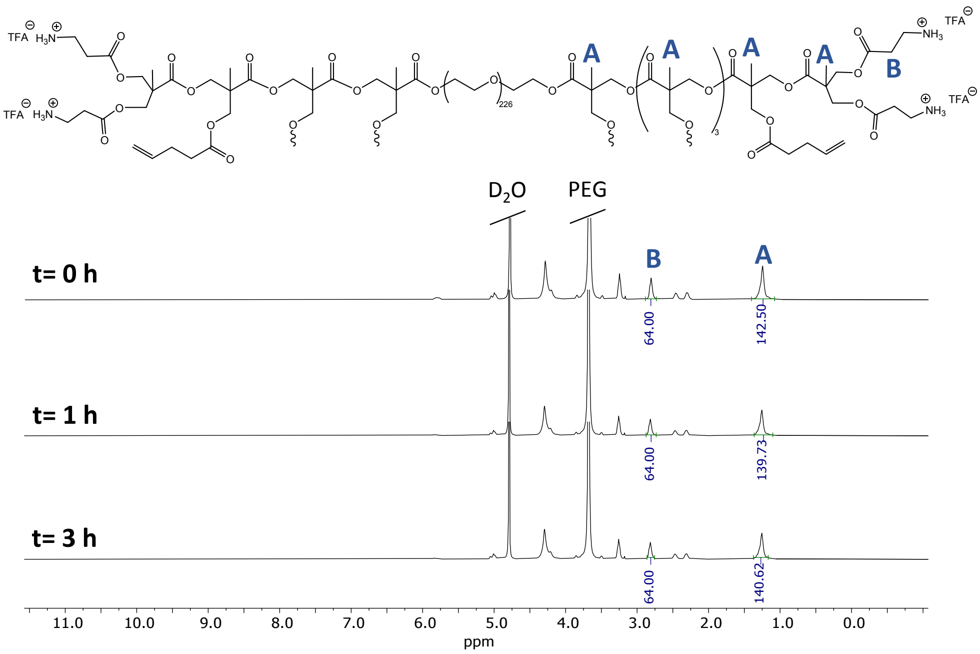

Figure S6. Stability of PEG10k-G4-(allyl)$_{16}$(NH_3^+)$_{32}$ in D_2O up to 3 h monitored by ^{1}H-NMR.

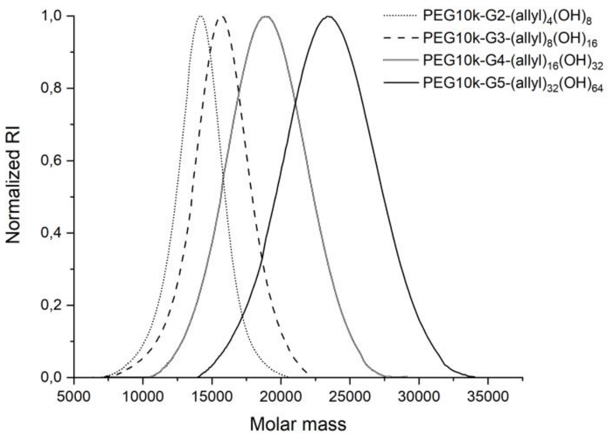

Figure S7. SEC analysis of PEG10K-Gn-(allyl)$_m$(OH)$_{2m}$ where 2<n<5 and 4<m<32.

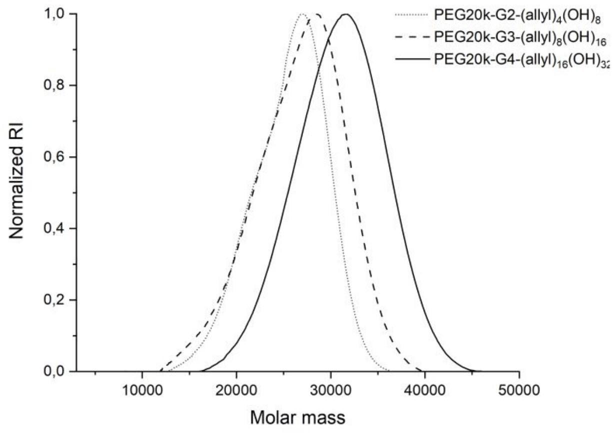

Figure S8. SEC analysis of PEG20K-Gn-(allyl)$_m$(OH)$_{2m}$ where 2<n<4 and 4<m<16.

	E. coli 178 **(Gram-(-))**				***S. aureus 2569*** **(Gram-(+))**			
	MIC		MBC		MIC		MBC	
Compound	*µg/ml*	*µM*	*µg/ml*	*µM*	*µg/ml*	*µM*	*µg/ml*	*µM*
PEG**10K**-**G3**-**(allyl)**$_8$**(OH)**$_{16}$	> 5000	> 378.5	> 5000	> 378.5	> 5000	> 378.5	> 5000	> 378.5
PEG**10K**-**G4**-**(allyl)**$_{16}$**(OH)**$_{32}$	> 5000	> 300.0	> 5000	> 300.0	> 5000	> 300.0	> 5000	> 300.0
PEG**20K**-**G4**-**(allyl)**$_{16}$**(OH)**$_{32}$	> 5000	> 190.0	> 5000	> 190.0	> 5000	> 190.0	> 5000	> 190.0
PEG**10K**-**G5**-**(allyl)**$_{32}$**(OH)**$_{64}$	> 5000	> 221.4	> 5000	> 221.4	> 5000	> 221.4	> 5000	> 221.4
PEG**10K**-**G3**-**(NH$_3$)**$_{16}$	> 5000	> 342.8	> 5000	> 342.8	-	-	-	-
PEG**20K**-**G4**-**(NH$_3$)**$_{32}$	625	21.3	625	21.3	-	-	-	-
**Cysteamine HCl**	> 5000	4×10^4	> 5000	4×10^4	> 5000	4×10^4	> 5000	4×10^4

Figure S9. Minimal Inhibitory concentration (MIC) and Minimal Bactericidal concentration (MBC) towards *E. coli 178 and S. aureus 2569* of hydroxyl heterofunctionalized precursors (PEG-Gn-(allyl)$_m$(OH)$_{2m}$ where 3<n<5 and 8<m<32), homofunctionalized DLDs PEG10k-G3-(NH_3^+)$_{16}$ and PEG20k-G4-(NH_3^+)$_{32}$ and cysteamine HCl.

DLD	Ratio (allyl : SH)	Dry content (% wt.)
PEG**10k**-**G3**-(allyl)$_8$(NH$_3^+$)$_{16}$	1:1	10
PEG**10k**-**G4**-(allyl)$_{16}$(NH$_3^+$)$_{32}$		
PEG**20k**-**G4**-(allyl)$_{16}$(NH$_3^+$)$_{32}$		
PEG**10k**-**G5**-(allyl)$_{32}$(NH$_3^+$)$_{64}$		
PEG**10k**-**G3**-(allyl)$_8$(NH$_3^+$)$_{16}$	1:1	20
PEG**10k**-**G4**-(allyl)$_{16}$(NH$_3^+$)$_{32}$		
PEG**20k**-**G4**-(allyl)$_{16}$(NH$_3^+$)$_{32}$		
PEG**10k**-**G5**-(allyl)$_{32}$(NH$_3^+$)$_{64}$		
PEG**10k**-**G3**-(allyl)$_8$(NH$_3^+$)$_{16}$	1:0.5	20
PEG**10k**-**G4**-(allyl)$_{16}$(NH$_3^+$)$_{32}$		
PEG**20k**-**G4**-(allyl)$_{16}$(NH$_3^+$)$_{32}$		
PEG**10k**-**G5**-(allyl)$_{32}$(NH$_3^+$)$_{64}$		
PEG**10k**-**G3**-(allyl)$_8$(NH$_3^+$)$_{16}$	1:0.25	20
PEG**10k**-**G4**-(allyl)$_{16}$(NH$_3^+$)$_{32}$		
PEG**20k**-**G4**-(allyl)$_{16}$(NH$_3^+$)$_{32}$		
PEG**10k**-**G5**-(allyl)$_{32}$(NH$_3^+$)$_{64}$		
PEG**10k**-**G4**-(allyl)$_{16}$(NH$_3^+$)$_{32}$	1:0.125	20
PEG**10k**-**G4**-(allyl)$_{16}$(NH$_3^+$)$_{32}$	1:0.063	20
PEG**10k**-**G4**-(allyl)$_{16}$(NH$_3^+$)$_{32}$	1:0.25	30

Figure S10. Tested formulations for hydrogels at different ratios allyl: SH$_{cross}$ and dry contents using DLDs-AB$_2$C and PEG2k-SH crosslinker. All formulations have been prepared using LAP as photoinitiator (2.4 % wt.) with a final volume of 50 µL.

A)

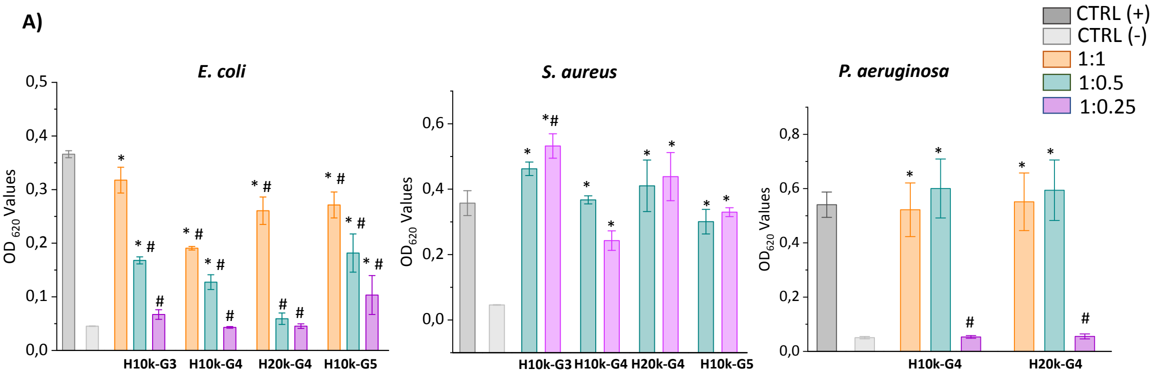

B)

Hydrogel	Ratio (allyl:SH$_{cross}$)	Moles NH$_3^+$ per hydrogel	Bactericidal effect		
			E. coli	*S. aureus*	*P. aeruginosa*
**H10K-G3**	1:0.25	0.8	No	No	-
**H10K-G4**	1:0.25	1.4	Yes	No	Yes
**H20K-G4**	1:0.25	1.0	Yes	No	No
**H10K-G5**	1:0.25	1.2	Yes	No	-

Figure S11. A) Screening of antibacterial activity in solution of hydrogels at 10^4 CFU/mL of *E. coli 178*, *P. aeruginosa 22.644* and *S. aureus 2569* compared to untreated bacteria solution

(CTRL (+)) and culture medium (CTRL (-)). B) Bactericidal evaluation for hydrogels that showed similar OD_{620} values to culture medium. (-) Formulation that has not been tested. Statistical analysis: One-way ANOVA (Bonferroni). # p<0,5 vs CTRL (+); * p<0,5 vs CTRL (-), n=3.

	**Dry conditions**		**Wet conditions**	
**Specimen**	Maximum Load [N]	Tensile stress [KPa]	Maximum Load [N]	Tensile stress [KPa]
**1**	15.49739	61.98957	7.80048	31.20190
**2**	15.09226	60.36904	7.58813	30.35254
**3**	15.79146	63.16584	6.42066	25.68265
**4**	14.24036	56.96145	6.66651	26.66605
**5**	15.90973	63.63893	*4.65497**	*18.61987**
**Average ± SD**	**15.3 ± 0.6**	**61.2 ± 2.4**	**7.1 ± 20.6**	**28.5 ± 2.3**

Figure S12. Maximum load and tensile stress values for five independent specimens (n=5). *Atypical result which was excluded as an outlier and not included in the calculation of the average and standard deviation (SD).

A)

**Hydrogel**	**Crosslinker**	**Ratio allyl:SH_{cross}:SH_{cys}**	***E. coli*** Diameter (cm)	***P. aeruginosa*** Diameter (cm)	***S. aureus*** Diameter (cm)
H10k-G4	PEG2k-SH	1:0.25:0	0.6 ± 0.0	0.6 ± 0.0	0
		1:0.25:0.75	0.7 ± 0.0	0.73 ± 0.04	0
H20k-G4		1:0.25:0	0.7 ± 0.0	0.7 ± 0.0	0
		1:0.25:0.75	0.63 ± 0.04	0.7 ± 0.0	0

B)

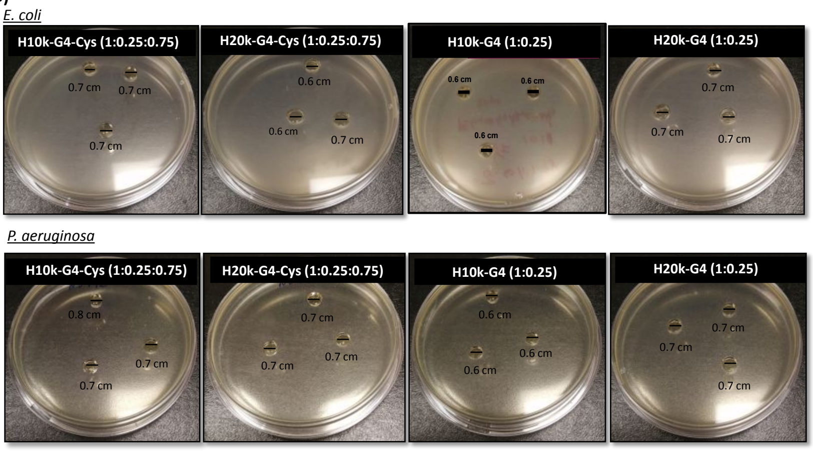

Figure S13. Antibacterial activity of hydrogels obtained from disk diffusion method at 10^6 CFU/mL of *E. coli 178*, *P. aeruginosa 22.644* and *S. aureus 2569*. A) Values of diameters obtained are summarized on the table (n=3). For *S. aureus*, significant decrease of bacterial growth was observed but still bacteria grew so no diameters are reported. B) Pictures of the agar plates.

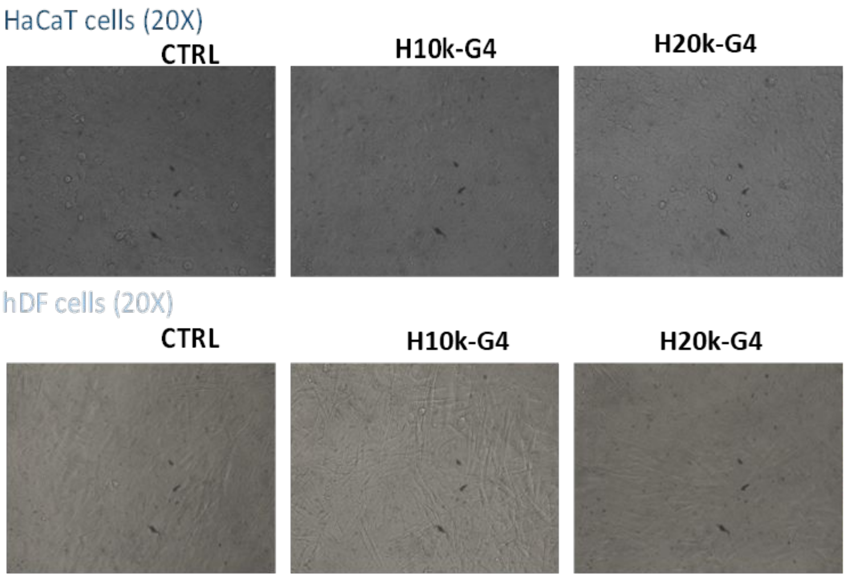

Figure S14. Captured images of Human keratinocytes (HaCaT) and Human Dermal Fibroblasts (hDF) after the treatment with the leach-out from the hydrogels H10k-G4 and H20k-G4 with PEG2k-SH as crosslinker. Ratio of the formulations (allyl: SH_{cross}, 1:0.25). Magnification 20X.

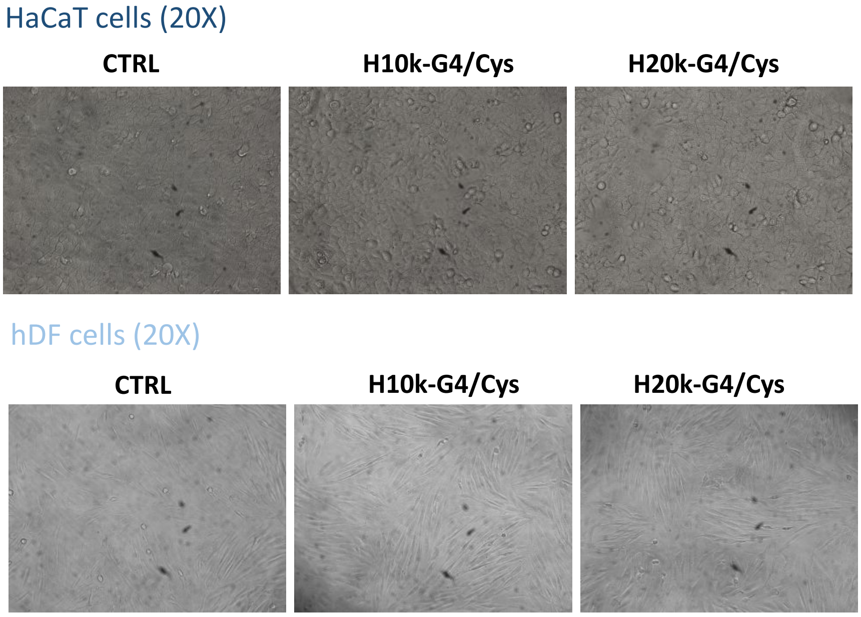

Figure S15. Captured images of Human keratinocytes (HaCaT) and Human Dermal Fibroblasts (hDF) after the treatment with the leach-out from the hydrogels H10k-G4/Cys and H20k-G4/Cys using PEG2k-SH as crosslinker. Ratio of the formulations (allyl: SH_{cross}: SH_{cys}, 1:0.25:0.75). Magnification 20X.

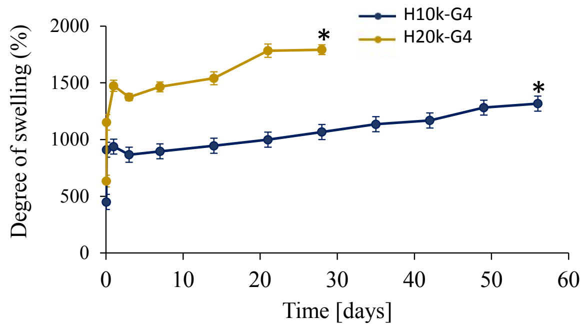

Figure S16. Swelling degree for the hydrogels (20 %wt) based on PEG10k and PEG20k of fourth generation crosslinked with PEG2kSH on a ratio 1:0.25 (n=5). * Degraded

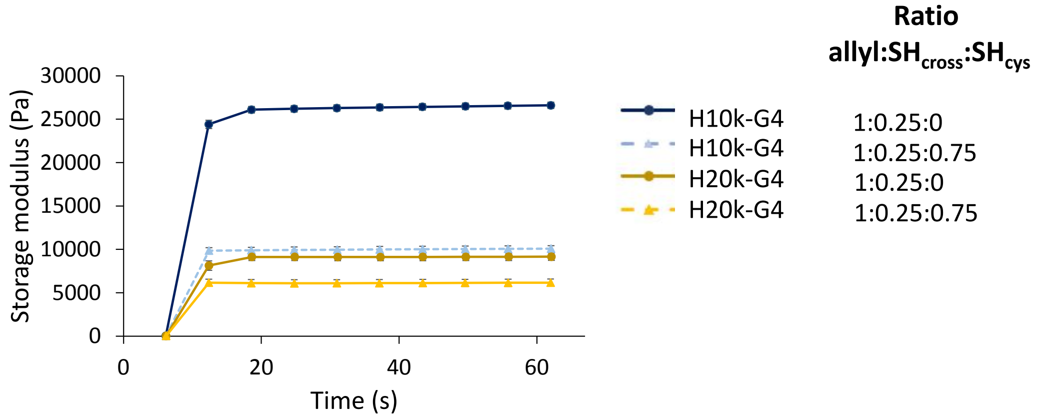

Figure S17. Time sweep experiments performed at room temperature on the formulations (20 %wt) with and without cysteamine using the UV curing accessory from TA Instruments (n=4).

References

1. García-Gallego, S.; Stenström, P.; Mesa-Antunez, P.; Zhang, Y.; Malkoch, M., *Biomacromolecules* **2020,** *21* (10), 4273-4279.

2. Stenstrom, P.; Hjorth, E.; Zhang, Y.; Andrén, O. C.; Guette-Marquet, S.; Schultzberg, M.; Malkoch, M., *Biomacromolecules* **2017,** *18* (12), 4323-4330.

3. Öberg, K.; Hed, Y.; Rahmn, I. J.; Kelly, J.; Löwenhielm, P.; Malkoch, M., *Chemical Communications* **2013,** *49* (62), 6938-6940.

4. Andrén, O. C.; Ingverud, T.; Hult, D.; Håkansson, J.; Bogestål, Y.; Caous, J. S.; Blom, K.; Zhang, Y.; Andersson, T.; Pedersen, E., *Advanced healthcare materials* **2019,** *8* (5), 1801619.
